# Optimizing Grape Quality Through Tillage and Organic Fertilization: A Comprehensive Analysis of Phenolic and Anthocyanin Variability Over Three Years

**DOI:** 10.1002/fsn3.4500

**Published:** 2024-10-10

**Authors:** Ozkan Kaya, Fadime Ates, Selda Daler, Sevil Canturk, Metin Turan, Harlene Hatterman‐Valenti

**Affiliations:** ^1^ Erzincan Horticultural Research Institute Republic of Türkiye Ministry of Agriculture and Forestry Erzincan Turkey; ^2^ Department of Plant Sciences North Dakota State University Fargo North Dakota USA; ^3^ Department of Life Sciences WesternCaspian University Baku Azerbaijan; ^4^ Manisa Viticulture Research Institute Republic of Türkiye Ministry of Agriculture and Forestry Manisa Turkey; ^5^ Department of Horticulture, Faculty of Agriculture Yozgat Bozok University Yozgat Turkey; ^6^ Department of Horticulture, Faculty of Agriculture Çukurova University Adana Turkey; ^7^ Faculty of Economy and Administrative Science Yeditepe University Istanbul Turkey

**Keywords:** crop yield, grapevine, organic fertilization, tillage practices, *Vitis* spp.

## Abstract

Knowing the concentration levels of phenolic compounds and anthocyanins in grape berries plays a key role, as these compounds significantly contribute to the nutritional value and quality of grapes, affecting both health benefits and grape quality. The current study was conducted to investigate how different tillage (disc harrow, chisel, no tillage) and organic fertilizer (Antep radish and broccoli chopped residue, olive blackwater) treatments affect the concentration of these valuable compounds in Royal grape berries over three consecutive years (2020–2022), providing insights into optimal agricultural practices for enhancing grape quality. Our results documented significant variations in phenolic and anthocyanin concentrations across different soil tillage, organic fertilizer treatments and years. Among the soil treatments, the disc harrow treatment emerged as the most effective in increasing the levels of most phenolic compounds and anthocyanins, while the broccoli fertilizer treatment was identified as the superior fertilizer application for achieving the same goal. Excluding disc harrow and broccoli fertilizer treatments, other treatments such as no tillage and chisel tillage, and fertilizers like olive blackwater and Antep radish, showed variable effectiveness. These treatments, although less effective overall than the leading treatments, contributed to increased levels of certain compounds such as resveratrol and pterostilbene, indicating their specific benefits. The findings also indicated specific combinations of tillage and fertilizer treatments that maximized the accumulation of certain phenolic compounds, like the combination of no tillage with broccoli fertilizer, which was particularly effective in increasing the levels of vanillic acid and *trans*‐caffeic acid. To sum up, by adopting practices such as disc harrow tillage and broccoli fertilizer application, viticulturists can enhance the phenolic and anthocyanin profiles of grapes, thus improving their nutritional value and potentially the quality of grape and wine produced from these grapes.

## Graphical abstract

Disc harrow tillage and broccoli fertilizer application significantly increased phenolic compounds and anthocyanins in Royal grapes over 3 years. Specific tillage‐fertilizer combinations optimized particular compounds. The results suggested viticulturists can enhance grape nutritional value and quality through these practices.

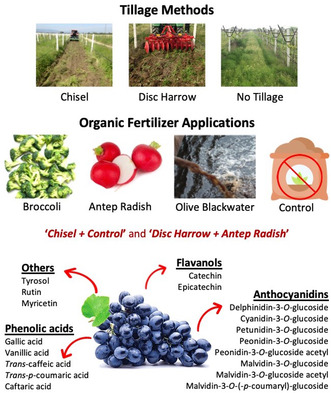

## 1 Introduction

The intensity and frequency of tillage practices significantly influence the soil biota within arable agroecosystems (Ivask, Kuu, and Sizov 2007; Capowiez, Cadoux, Bouchand, Roger‐Estrade, et al. 2009; Capowiez, Cadoux, Bouchand, Ruy, et al. 2009). In the context of perennial agroecosystems like vineyards, it has been established that tillage activities contribute to a reduction in both plant and animal diversity (Kazakou et al. 2016). Moreover, practices associated with tillage and non‐chemical weed management strategies (including harrowing and mulching), alongside mineral application and various other agricultural interventions, have been shown to impact soil functionality to different degrees (Ivask, Kuu, and Sizov 2007; Capowiez, Cadoux, Bouchand, Roger‐Estrade, et al. 2009; Capowiez, Cadoux, Bouchand, Ruy, et al. 2009). Farmers' soil management practices in the spaces between vine rows in vineyards are influenced by various factors, as indicated by Steenwerth and Belina (2008). These include long‐standing viticultural practices, specific soil and climate conditions of the region, the slope of the vineyards, types of machinery used, and often personal preferences. In particular, soil erosion emerges as a serious issue in vineyards located on slopes, with gravelly soils and low organic matter content; this situation has been detailed in studies by Ruiz‐Colmenero, Bienes, and Marques (2011) and Martínez‐García et al. (2018), emphasizing the importance of sustainable management strategies. Traditional soil working methods, like using a moldboard plow, have been criticized for causing soil erosion, damaging the structure of soil aggregates, and leading to significant mineral losses (Karlen et al. 2013). In response, reduced or no tillage methods have been proposed. These methods encourage coverage of at least 30% of the soil surface with plant residues after planting, which has been noted to reduce soil physical disturbance (Liu et al. 2014) and increase the soil's carbon storage capacity (Shrestha et al. 2015). However, there are conflicting results suggesting that conservation tillage may reduce soil carbon levels compared to conventional methods, possibly owing to less carbon being incorporated into the soil due to the retention of plant residues on the soil surface or a reduced production of plant residues (Angers et al. 1997; Vanden Bygaart et al. 2002). Additionally, changes in tillage methods have led to significant changes in the composition and decomposition of soil organic matter (Shrestha et al. 2015; Pisani et al. 2016). No tillage methods reduce the amount of lipid components (alkyl carbon) while increasing the proportion of cellulose‐type components (O‐alkyl carbon) compared to conventional tillage. This leads to changes in microbial processing and decomposition, resulting in a lower stage of organic matter decomposition in the upper soil layer (0–10 cm) owing to changes in microbial community composition and diversity (Shrestha et al. 2015). Compared to traditional methods, eliminated tillage or systems with reduced have a higher fungus‐to‐bacteria ratio (Panettieri et al. 2020) and greater bacterial diversity (De Quadros et al. 2012). These differences have been attributed to increases in microbial abundance owing to changes in the availability of substrates and the biological and physical environment (Ghimire et al. 2014; Wang et al. 2014; Lupwayi et al. 2017).

The European Union (EU) utilizes geographical schemes like Protected Geographical Indication (PGI), Protected Designation of Origin (PDO), and Traditional Specialties Guaranteed (TSG) to show and protect high‐quality agricultural products on a global scale. These labels are based on unique regional features, including soil and climate conditions, grape varieties, farming practices, and cultural heritage. Such designations help promote products with a specific geographical origin and possess qualities or a reputation owing to that origin (Kaya, Ates, et al. 2024; Kaya, Yilmaz, et al. 2024). In the rapidly evolving sector of viticulture, which has shown notable progress and the integration of advanced technologies, there's a concerted push toward improving both the quality and quantity of grapes. However, this drive for enhancement often leads to the overuse of soil resources, pivotal for grape cultivation. The potential reduction in soil fertility stems from a complex interplay of factors, including limited use of organic fertilizers, intensive agricultural mechanization characterized by frequent machinery use on the soil, and widespread application of agrochemicals (Souri and Hatamian 2019). Such practices, while aimed at boosting production, may inadvertently compromise the sustainability of soil health and, by extension, the long‐term viability of viticulture in these geographically protected areas. In this regard, the growing interest in organic agriculture for food production globally is largely driven by the belief that consuming organic foods offers health benefits (Johansson et al. 2014). Organic fertilization offers a sustainable approach to address the adverse environmental effects of chemical fertilizers while maintaining improved and long‐lasting soil health and plant productivity (Döring et al. 2015; Souri and Hatamian 2019). Organic manures not only improve soil's physical, chemical, and biological properties but also enhance the efficiency of chemical fertilizers when applied either alone or in combination with pellet fertilizers (Souri, Rashidi, and Kianmehr 2018). Originally, organic farming aimed to preserve and boost the health of soil, plants, animals, and humans. Integrated fertilization combines the benefits standing at the midpoint between traditional and organic methods. While chemical fertilizers have significantly boosted global crop yields, concerns about their overuse, damaging beneficial microorganisms, contaminating soil, impacting human health, and harming environmental quality have been raised (Patra et al. 2019). Integrated systems aim to sustain crop yields with minimal synthetic chemical residues, thus reducing the unwanted effects of chemical fertilizers' overuse (Lososová et al. 2011).

The synthesis of polyphenols in grape berries is a complex process, largely determined by the plant's genetic makeup; however, the diversity and abundance of phenolic compounds in grapes can be affected by several key factors: the characteristics of the grape itself, such as the variety and ripening level at the time of harvest; the techniques used in vine cultivation; and environmental conditions, including soil type and climate as well as practices related to tillage and fertilization also play a significant role (Obreque‐Slier et al. 2010). Some studies into how vineyards are tilled and using organic fertilizers indicate how these methods may change the way grape berries develop their phenolic compounds, which are important for the berry's taste and color (Kaya, Ates, et al. 2024; Kaya, Yilmaz, et al. 2024). The effects of tillage and organic fertilization on vineyards are complex, varying with multiple factors. Understanding their general impact on vine growth and grape quality, regardless of specific conditions, could provide valuable guidelines for vineyard management. We conducted a 3‐year study on Royal grape vineyards, examining three tillage strategies (chisel, disc harrow, no tillage) and three organic fertilization treatments (olive blackwater, Antep radish, broccoli compost). The purposes of our study were (i) to assess the effects of different tillage management strategies on berry phenolic compounds and grape quality; (ii) to evaluate the impact of various organic fertilization treatments on vine health and grape characteristics; (iii) to identify consistent reactions of grapevines to these management practices across different conditions; and (iv) to contribute new insights to the existing knowledge on vineyard management, particularly regarding phenolic compounds.

## 2 Materials and Methods

### 2.1 Plant Selection and Experimental Site

The study was conducted at the Manisa Viticulture Research Institute, located at 27°23′59.43″ E, 38°38′0.9.40″ N, from 2020 to 2022. Manisa province, situated just 41 km from the Aegean Sea, lies between 27°08′ and 29°05′ east longitudes and 38°04′ and 39°58′ north latitudes, covering an area of 13,810 km^2^. The prevailing climate in Manisa is classified as a Mediterranean‐type land climate. This region experiences rising temperatures during the summer months and increased rainfall during the winter. Summer in Manisa is characterized by very hot weather, typical of the continental Mediterranean climate. The average annual temperature is 16.3°C, with January and February being the coldest months. Western Anatolia, where Manisa is located, exhibits the precipitation patterns characteristic of the Mediterranean climate (MMMID 2023). Soil samples were collected from a depth of 0 to 30 cm, and a series of physical and chemical analyses were conducted to determine the initial soil properties. The cation exchange capacity (CEC) was measured using sodium acetate buffered at pH 8.2, while exchangeable cations were determined with ammonium acetate buffered at pH 7 (Sumner and Miller 1996; Thomas 1982). The Kjeldahl method was used to assess organic nitrogen (Bremner 1996), and plant‐available phosphorus (P) was quantified using the sodium bicarbonate method (Olsen et al. 1954). Electrical conductivity (EC) was measured in saturation extracts following Rhoades (1996), and soil pH was determined in a 1:2 soil‐to‐water extract ratio. Calcium carbonate (lime) concentrations were assessed according to McLean (1982), while soil organic matter was determined using the Smith Weldon method (Nelson and Sommers 1982). The concentrations of phosphorus (P), potassium (K), calcium (Ca), magnesium (Mg), iron (Fe), zinc (Zn), manganese (Mn), and copper (Cu) were determined using an inductively coupled plasma spectrophotometer. The results of the trial 3 vineyard showed that soil saturation increased with depth, ranging from 32.99% at 0 to 20 cm to 36.08% at 60 to 90 cm, before decreasing to 30.93% at 90 to 120 cm. The soil was consistently loamy across all depths. Salt concentration remained low, ranging from 0.0055 to 0.0080 dS m^−1^, while pH values increased slightly with depth, from 7.67 at 0 to 20 cm to 7.96 at 90 to 120 cm. Lime content showed a gradual increase with depth, from 4.88% at 0 to 20 cm to 8.95% at 90 to 120 cm. Organic matter content was highest at the surface (1.61% at 0–20 cm) and decreased with depth, reaching 0.54% at 90–120 cm. Regarding nutrient content, phosphorus (P) levels decreased with depth, from 5.66 ppm at 0 to 20 cm to 2.24 ppm at 90 to 120 cm. Potassium (K) levels also declined significantly with depth, from 81.88 ppm at 0 to 20 cm to 9.98 ppm at 90 to 120 cm. Calcium (Ca) content increased with depth, peaking at 4572 ppm at 40 to 60 cm, before decreasing to 3240 ppm at 90 to 120 cm. Magnesium (Mg) content followed a similar pattern, increasing from 141.4 ppm at 0 to 20 cm to 170.4 ppm at 60 to 90 cm, before decreasing to 129.8 ppm at 90 to 120 cm. The concentrations of iron (Fe), zinc (Zn), manganese (Mn), and copper (Cu) also exhibited variability with depth, generally decreasing as depth increased.

The experiment included ‘Royal’ grapes grafted onto 110 R rootstock (*V. berlandieri* × *V. rupestris*) in 2012. The ‘Royal’ grape cultivar is known for its large, winged, slightly flattened round, conical clusters (400–500 g) and large, purple‐black berries (each approximately 8 g) with a mild tannic taste. These vines were trained on a Y‐shaped trellis system with 3 × 2 m spacing, using a double training system and short pruning to leave 2–3 buds per cane. Managed through short pruning to control growth and maximize cluster yield, this cultivar demonstrates strong growth with yields ranging from 1990 to 2325 kg da^−1^. Harvests were carried out from the end of August to September to ensure optimum berry ripening. The trial design was a randomized complete block with four replications, each containing 12 vines, and used drip irrigation for efficient water distribution to the roots.

### 2.2 Soil Management Techniques

The experiment utilized various tillage implement materials to assess their effects on soil and crop performance. The machinery used included a plow with four bodies, a working width of 1.35 m, and a tillage depth of 18 cm. This implement was used for primary tillage, inverting the soil and preparing the seedbed. A disk harrow with 18 discs, covering a working width of 1.9 m and operating at a depth of 15 cm, was utilized to break down soil clods and incorporate residues. A chisel, featuring seven legs and a working width of 2.1 m, was used for deep tillage at a depth of 21 cm, loosening the soil while preserving its structure. Additionally, a heavy‐duty disk harrow with 20 discs, a working width of 2.0 m, and a tillage depth of 17 cm was used for more intensive soil mixing and residue management. The study utilized three different soil tillage techniques applied between vine rows. The chisel method, applied in Autumn and April, involved loosening the soil without turning it over. Disc harrowing was also used to break and aerate the soil in Autumn and April. Control vines received no soil tillage, serving as a comparison for the effects of soil management.

### 2.3 Organic Fertilization Application

The effects of organic chopped residue fertilizers: broccoli (*Brassica oleracea* L. var. Italica), Antep radish (*Raphanus raphanistrum* L.), and olive blackwater on soil quality were investigated. Olive blackwater consists of the sap inside the olives, which is produced during the conversion of olives into olive oil, olive washing water, water added during the process and water leaking from the pomace. Broccoli and Antep radish roots, leaves and edible parts were harvested from a different field and converted into shredded and chopped residue. These were selected for their high organic matter and mineral content, aiming to improve soil health and sustainability. Olive blackwater, containing various components from olive processing, was sourced from a specialized production company. In the second week of April, following soil tillage, each vine was treated between 08:00 and 10:00 AM with 6 kg of crushed broccoli and 6 kg of crushed Antep radish, along with 6 L of 25% olive blackwater solution. The olive blackwater was applied using a fertilizer tank, while the broccoli and Antep radish were shredded with a chopper and then applied to cover both the inter‐row and row surfaces.

### 2.4 Sample Collection

‘Royal’ grape clusters (17° Brix) were harvested in August, and 30 clusters from each treatment group were randomly selected. 100 berry samples with intact stems were collected from these clusters to prevent juice loss. Samples were then transported to the laboratory in plastic bags, and 50 g of berry samples for biochemical analysis was repeated three times. Upon arrival at the laboratory, grape clusters were promptly stored at 4°C and subsequently frozen at −80°C for subsequent analysis.

### 2.5 Quantification of Phenolic Compounds in Grape Berries by HPLC

Berry samples from the ‘Royal’ grape cultivar were analyzed for their phenolic content. The preparation process involved grinding the whole berry samples from the clusters into a uniform mixture using a standard blender. The phenolic compounds assessed included *trans*‐caffeic acid, vanillic acid, gallic acid, ferulic acid, *trans‐p*‐coumaric acid, caftaric acid, resveratrol, pterostilbene, piceid, viniferin, tyrosol, catechin, epicatechin, rutin, quercetin and myricetin. Adapting a method by Cosme, Pinto, and Vilela (2018) for extracting phenolic compounds and doing it three times, we first mixed the crushed grape samples with an equal amount of distilled water and then spun them in a centrifuge at 15,000 rpm for 15 min. After that, we filtered the liquid part (supernatant) through very fine filters (0.45 μm) and analyzed it with a High‐Performance Liquid Chromatography (HPLC) setup. We used an Agilent 1100 HPLC machine with a special detector (diode‐array) and a column made of octadecyl‐silica for this. To separate the compounds, we passed a mixture of methanol, water, and acetic acid through the column in two different ratios: one with more water (component A: 10% methanol, 28% water, 2% acetic acid) and one with more methanol (component B: 90% methanol, 8% water, 2% acetic acid). We set the machine to look for the compounds at specific light wavelengths (254 and 280 nm), injected 20 μL of the sample each time, and kept the liquid moving through the system at a rate of 1 mL min^−1^. For looking specifically at anthocyanins, which are compounds that give berries their color and include types like petunidin‐3‐*O*‐glucoside and malvidin‐3‐*O*‐glucoside, we used a method inspired by El Youssef et al. (2012). This involved the same HPLC setup but a mass spectrometer was added to identify the compounds more accurately. We injected the anthocyanin extracts into a special column (Zorbax Eclipse XDB‐C18) and used a mix of water, acetonitrile, and formic acid to separate them. The process gradually changed the mix of these liquids over 36 min to catch all the different types of anthocyanins, starting with mostly water and a little acetonitrile and formic acid, then moving to an even mix of water and acetonitrile, and finally to mostly acetonitrile, before going back to the starting conditions. The column was maintained at 40°C, with a flow rate of 0.19 mL min^−1^ and an 8‐min conditioning period between injections.

### 2.6 Data Analysis

All experiments were conducted using a Factorial Randomized Complete Block Design (Factorial RCD). Quantitative data showcasing the effects of the main applications (years, soil management, and fertilization) and their interactions on phenolic compounds were subjected to an extensive variance analysis (three‐way ANOVA) using IBM SPSS Statistics V22.0. Duncan's Multiple Range Test, with a significance threshold of *p ≤* 0.0001, was used to distinguish significant differences between means. Data were presented as mean values along with the corresponding standard error (SE). Detailed data for all treatments were provided in the Data S1 as mean and standard deviation (SD). The relationships between factors were examined using Principal Component Analysis (PCA), generated by GraphPad Prism version 9.3.1 (GraphPad Software, LLC, San Diego, CA, USA), and the results were defined using a biplot (Evgenidis, Traka‐Mavrona, and Koutsika‐Sotiriou 2011). A hierarchical clustering heatmap was also created to visualize the relationships and intensities between the factors and the studied properties, using the SRPLOT online platform (https://www.bioinformatics.com.cn, accessed on March 10, 2024).

## 3 Results

A 3‐year study (2020–2022) on ‘Royal’ grape berries revealed significant variations in phenolic compounds and anthocyanins across years, tillage methods, and fertilizer treatments (Table 1). The 2022 season showed notably lower concentrations of several compounds, including caftaric acid (4.64 μg g^−1^ FW), tyrosol (5.37 μg g^−1^ FW), epicatechin (2.77 μg g^−1^ FW), and myricetin (1.67 μg g^−1^ FW), as well as reduced levels of various anthocyanins such as delphinidin‐3‐*O*‐glucoside (2.93%). Throughout the study period, peonidin‐3‐*O*‐glucoside acetyl, malvidin‐3‐*O*‐glucoside, and peonidin‐3‐*O*‐glucoside emerged as the most abundant compounds, with average concentrations of approximately 46.45%, 45.16%, and 34.02%, respectively. Conversely, pterostilbene, myricetin, and cyanidin‐3‐*O*‐glucoside were found in the lowest concentrations, averaging around 1.20, 1.84, and 1.88 μg g^−1^ FW, respectively. Notably, the research identified disc harrow tillage as the most effective method for enhancing the levels of various phenolic compounds and anthocyanins in the grape berries, underscoring the importance of cultivation practices in influencing berry composition. The study on ‘Royal’ grapes revealed significant variations in phenolic compounds and anthocyanins across different tillage and fertilizer treatments. Disc harrow tillage yielded the highest levels for most compounds, including gallic acid (3.01 μg g^−1^), vanillic acid (4.45 μg g^−1^), *trans*‐caffeic acid (3.42 μg g^−1^), *trans*‐*p*‐coumaric acid (4.41 μg g^−1^), caftaric acid (5.44 μg g^−1^), viniferin (14.67 μg g^−1^), tyrosol (6.55 μg g^−1^), catechin (5.83 μg g^−1^), epicatechin (3.28 μg g^−1^), and rutin (2.91 μg g^−1^). This treatment also showed the highest anthocyanin levels, such as delphinidin‐3‐*O*‐glucoside (3.58%), cyanidin‐3‐*O*‐glucoside (2.43%), petunidin‐3‐*O*‐glucoside (7.11%), peonidin‐3‐*O*‐glucoside (45.92%), peonidin‐3‐*O*‐glucoside acetyl (60.34%), malvidin‐3‐*O*‐glucoside (56.27%), malvidin‐3‐*O*‐glucoside acetyl (34.47%), and malvidin‐3‐*O*‐(−*p*‐coumaryl)‐glucoside (4.63%). The no tillage treatment exhibited the highest levels of ferulic acid (3.42 μg g^−1^), resveratrol (12.73 μg g^−1^), pterostilbene (1.31 μg g^−1^), quercetin (3.29 μg g^−1^), and myricetin (2.04 μg g^−1^). Broccoli fertilizer treatment emerged as the best for enhancing various phenolic compounds and anthocyanins, with highest levels of gallic acid (2.77 μg g^−1^), vanillic acid (4.58 μg g^−1^), *trans*‐caffeic acid (3.39 μg g^−1^), ferulic acid (3.01 μg g^−1^), *trans*‐*p*‐coumaric acid (4.29 μg g^−1^), caftaric acid (5.32 μg g^−1^), viniferin (14.54 μg g^−1^), tyrosol (6.42 μg g^−1^), catechin (5.98 μg g^−1^), and epicatechin (3.39 μg g^−1^). Olive blackwater treatment showed highest levels of resveratrol (11.12 μg g^−1^), quercetin (3.12 μg g^−1^), malvidin‐3‐*O*‐glucoside (50.46%), and petunidin‐3‐*O*‐glucoside (6.09%). The control treatment had the highest pterostilbene (1.44 μg g^−1^). Antep radish treatment showed higher levels of piceid (2.30 μg g^−1^) and malvidin‐3‐*O*‐(‐*p*‐coumaryl)‐glucoside (6.10%), but lower levels of myricetin (1.78 μg g^−1^), cyanidin‐3‐*O*‐glucoside (1.73%), and pterostilbene (1.05 μg g^−1^).

**TABLE 1:** Effect of fertilizer application on phenolic compounds (μg g^−1^ FW) in ‘Royal’ grape cultivar depending on soil management, fertilizer treatments and years of research.

Factors	Gallic acid	Vanillic acid	*Trans*‐caffeic acid	Ferulic acid	*Trans‐p*‐coumaric acid	Caftaric acid	Resveratrol	Pterostilbene	Piceid	Viniferin	Tyrosol	Catechin
Years (Y)^x^												
2020	2.61 ± 0.02 b	3.89 ± 0.03 b	2.69 ± 0.02 b	1.42 ± 0.02 b	3.95 ± 0.03 b	4.87 ± 0.03 a	3.86 ± 0.08 c	0.69 ± 0.00 c	1.03 ± 0.02 c	7.54 ± 0.05 c	6.18 ± 0.03 a	5.21 ± 0.05 b
2021	2.49 ± 0.02 c	3.85 ± 0.03 b	2.69 ± 0.02 b	1.48 ± 0.02 b	3.76 ± 0.03 c	4.95 ± 0.03 a	11.69 ± 0.08 b	1.19 ± 0.00 b	1.43 ± 0.02 b	12.21 ± 0.05 b	6.02 ± 0.03 b	5.09 ± 0.05 b
2022	2.74 ± 0.02 a	4.37 ± 0.03 a	3.54 ± 0.02 a	5.68 ± 0.02 a	4.14 ± 0.03 a	4.64 ± 0.03 b	15.78 ± 0.08 a	1.73 ± 0.00 a	3.24 ± 0.02 a	22.71 ± 0.05 a	5.37 ± 0.03 c	5.80 ± 0.05 a
Tillage (T)^y^												
Chisel	2.16 ± 0.02 c	3.65 ± 0.03 c	2.42 ± 0.02 c	1.83 ± 0.02 c	3.59 ± 0.03 c	3.99 ± 0.03 c	9.69 ± 0.08 b	1.25 ± 0.00 b	2.84 ± 0.02 a	13.38 ± 0.05 c	5.32 ± 0.03 c	4.98 ± 0.05 c
Disc Harrow	3.01 ± 0.02 a	4.45 ± 0.03 a	3.42 ± 0.02 a	3.32 ± 0.02 b	4.41 ± 0.03 a	5.44 ± 0.03 a	8.92 ± 0.08 c	1.06 ± 0.00 c	0.99 ± 0.02 c	14.67 ± 0.05 a	6.55 ± 0.03 a	5.83 ± 0.05 a
No Tillage	2.67 ± 0.02 b	4.01 ± 0.03 b	3.09 ± 0.02 b	3.42 ± 0.02 a	3.85 ± 0.03 b	5.03 ± 0.03 b	12.73 ± 0.08 a	1.31 ± 0.00 a	1.86 ± 0.02 b	14.40 ± 0.05 b	5.70 ± 0.03 b	5.29 ± 0.05 b
Fertilizer (F)^z^												
Olive Blackwater	2.65 ± 0.02 b	3.92 ± 0.04 c	2.89 ± 0.03 b	3.00 ± 0.03 a	3.92 ± 0.03 b	4.78 ± 0.04 b	11.12 ± 0.09 a	0.98 ± 0.00 d	2.27 ± 0.02 a	14.51 ± 0.06 a	6.07 ± 0.04 b	5.26 ± 0.05 b
Antep Radish	2.57 ± 0.02 c	4.04 ± 0.04 b	2.92 ± 0.03 b	2.69 ± 0.03 b	3.95 ± 0.03 b	4.80 ± 0.04 b	9.82 ± 0.09 c	1.05 ± 0.00 c	2.30 ± 0.02 a	13.30 ± 0.06 c	5.88 ± 0.04 c	5.38 ± 0.05 b
Broccoli	2.77 ± 0.02 a	4.58 ± 0.04 a	3.39 ± 0.03 a	3.01 ± 0.03 a	4.29 ± 0.03 a	5.32 ± 0.04 a	10.54 ± 0.09 b	1.35 ± 0.00 b	1.59 ± 0.02 b	14.54 ± 0.06 a	6.42 ± 0.04 a	5.98 ± 0.05 a
Control	2.44 ± 0.02 d	3.61 ± 0.04 d	2.70 ± 0.03 c	2.74 ± 0.03 b	3.64 ± 0.03 c	4.37 ± 0.04 c	10.30 ± 0.09 b	1.44 ± 0.00 a	1.43 ± 0.02 c	14.26 ± 0.06 b	5.05 ± 0.04 d	4.86 ± 0.05 c
Significance*												
Y	*F* = 47.449 *p* < 0.0001	*F* = 88.273 *p* < 0.0001	*F* = 437.683 *p* < 0.0001	*F* = 10,891.142 *p* < 0.0001	*F* = 55.564 *p* < 0.0001	*F* = 23.070 *p* < 0.0001	*F* = 6099.492 *p* < 0.0001	*F* = 71,364.676 *p* < 0.0001	*F* = 5359.672 *p* < 0.0001	*F* = 20,867.776 *p* < 0.0001	*F* = 151.315 *p* < 0.0001	*F* = 69.124 *p* < 0.0001
T	F = 558.949 *p* < 0.0001	*F* = 166.494 *p* < 0.0001	*F* = 469.689 *p* < 0.0001	*F* = 1455.538 *p* < 0.0001	*F* = 280.161 *p* < 0.0001	*F* = 483.159 *p* < 0.0001	*F* = 674.064 *p* < 0.0001	*F* = 4145.054 *p* < 0.0001	*F* = 3318.659 *p* < 0.0001	*F* = 159.480 *p* < 0.0001	*F* = 327.431 *p* < 0.0001	*F* = 88.532 *p* < 0.0001
F	*F* = 43.434 *p* < 0.0001	*F* = 128.043 *p* < 0.0001	*F* = 117.413 *p* < 0.0001	*F* = 40.539 *p* < 0.0001	*F* = 83.916 *p* < 0.0001	*F* = 97.662 *p* < 0.0001	*F* = 36.012 *p* < 0.0001	*F* = 9969.847 *p* < 0.0001	*F* = 587.363 *p* < 0.0001	*F* = 88.762 *p* < 0.0001	*F* = 210.599 *p* < 0.0001	*F* = 76.994 *p* < 0.0001
Y × T	*F* = 1170.904 *p* < 0.0001	*F* = 748.745 *p* < 0.0001	*F* = 913.991 *p* < 0.0001	*F* = 1293.543 *p* < 0.0001	*F* = 859.301 *p* < 0.0001	*F* = 1313.418 *p* < 0.0001	*F* = 3006.015 *p* < 0.0001	*F* = 12,177.169 *p* < 0.0001	*F* = 3451.510 *p* < 0.0001	*F* = 349.440 p < 0.0001	*F* = 1305.850 *p* < 0.0001	*F* = 525.662 *p* < 0.0001
Y × F	*F* = 120.235 *p* < 0.0001	*F* = 52.716 *p* < 0.0001	*F* = 81.965 *p* < 0.0001	*F* = 42.931 *p* < 0.0001	*F* = 76.000 *p* < 0.0001	*F* = 125.816 *p* < 0.0001	*F* = 199.846 *p* < 0.0001	*F* = 9780.739 *p* < 0.0001	*F* = 336.301 *p* < 0.0001	*F* = 71.752 *p* < 0.0001	*F* = 85.899 *p* < 0.0001	*F* = 35.653 *p* < 0.0001
T × F	*F* = 196.994 *p* < 0.0001	*F* = 93.705 *p* < 0.0001	*F* = 99.446 *p* < 0.0001	*F* = 75.485 *p* < 0.0001	*F* = 142.092 *p* < 0.0001	*F* = 158.616 *p* < 0.0001	*F* = 132.918 *p* < 0.0001	*F* = 9177.955 *p* < 0.0001	*F* = 500.978 *p* < 0.0001	*F* = 377.957 *p* < 0.0001	*F* = 311.888 *p* < 0.0001	*F* = 93.245 *p* < 0.0001
Y × T × F	*F* = 288.353 *p* < 0.0001	*F* = 275.636 *p* < 0.0001	*F* = 282.055 *p* < 0.0001	*F* = 62.722 *p* < 0.0001	*F* = 290.060 *p* < 0.0001	*F* = 362.953 *p* < 0.0001	*F* = 55.348 *p* < 0.0001	*F* = 15,234.624 *p* < 0.0001	*F* = 309.898 *p* < 0.0001	*F* = 143.583 *p* < 0.0001	*F* = 350.351 *p* < 0.0001	*F* = 195.606 *p* < 0.0001

Factors	Epicatechin (μg g^−1^ FW)	Rutin (μg g^−1^ FW)	Quercetin (μg g^−1^ FW)	Myricetin (μg g^−1^ FW)	Delphinidin‐3‐*O*‐glucoside (%)	Cyanidin‐3‐*O*‐glucoside (%)	Petunidin‐3‐*O*‐glucoside (%)	Peonidin‐3‐*O*‐glucoside (%)	Peonidin‐3‐*O*‐glucoside acetyl (%)	Malvidin‐3‐*O*‐glucoside (%)	Malvidin‐3‐*O*‐glucoside acetyl (%)	Malvidin‐3‐*O*‐(‐*p*‐coumaryl)‐glucoside (%)
Years (Y)^x^												
2020	3.03 ± 0.02 a	2.20 ± 0.01 b	2.18 ± 0.02 b	1.89 ± 0.02 b	3.52 ± 0.00 a	1.93 ± 0.01 b	6.82 ± 0.01 a	32.65 ± 0.09 b	60.24 ± 0.07 a	51.54 ± 0.09 a	40.39 ± 0.08 a	6.44 ± 0.02 a
2021	2.94 ± 0.02 b	2.20 ± 0.01 b	2.20 ± 0.02 b	1.97 ± 0.02 a	1.92 ± 0.00 c	1.21 ± 0.01 c	3.97 ± 0.01 c	17.77 ± 0.09 c	32.76 ± 0.07 c	32.26 ± 0.09 b	19.98 ± 0.08 c	3.51 ± 0.02 b
2022	2.77 ± 0.02 c	2.89 ± 0.01 a	4.45 ± 0.02 a	1.67 ± 0.02 c	2.93 ± 0.00 b	2.50 ± 0.01 a	5.93 ± 0.01 b	51.64 ± 0.09 a	46.35 ± 0.07 b	51.69 ± 0.09 a	21.22 ± 0.08 b	2.47 ± 0.02 c
Tillage (T)^y^												
Chisel	2.64 ± 0.02 c	1.88 ± 0.01 c	2.41 ± 0.02 c	1.55 ± 0.02 c	2.72 ± 0.00 b	1.60 ± 0.01 b	5.42 ± 0.01 b	21.24 ± 0.09 c	44.59 ± 0.07 b	43.11 ± 0.09 b	30.71 ± 0.08 b	4.23 ± 0.02 b
Disc Harrow	3.28 ± 0.02 a	2.91 ± 0.01 a	3.13 ± 0.02 b	1.95 ± 0.02 b	3.58 ± 0.00 a	2.43 ± 0.01 a	7.11 ± 0.01 a	45.92 ± 0.09 a	60.34 ± 0.07 a	56.27 ± 0.09 a	34.47 ± 0.08 a	4.63 ± 0.02 a
No Tillage	2.81 ± 0.02 b	2.49 ± 0.01 b	3.29 ± 0.02 a	2.04 ± 0.02 a	2.07 ± 0.00 c	1.60 ± 0.01 b	4.19 ± 0.01 c	34.90 ± 0.09 b	34.42 ± 0.07 c	36.12 ± 0.09 c	16.41 ± 0.08 c	3.57 ± 0.02 c
Fertilizer (F)^z^												
Olive Blackwater	2.79 ± 0.02 c	2.50 ± 0.02 b	3.12 ± 0.02 a	1.82 ± 0.02 b	2.96 ± 0.01 b	2.05 ± 0.01 a	6.09 ± 0.01 a	36.80 ± 0.11 b	50.47 ± 0.08 b	50.46 ± 0.10 a	29.12 ± 0.09 a	4.38 ± 0.02 b
Antep Radish	2.94 ± 0.02 b	2.39 ± 0.02 c	2.81 ± 0.02 c	1.78 ± 0.02 bc	2.51 ± 0.01 c	1.73 ± 0.01 b	5.21 ± 0.01 c	30.58 ± 0.11 c	42.20 ± 0.08 c	40.29 ± 0.10 c	26.30 ± 0.09 b	6.10 ± 0.02 a
Broccoli	3.39 ± 0.02 a	2.71 ± 0.02 a	3.01 ± 0.02 b	2.05 ± 0.02 a	3.20 ± 0.01 a	2.03 ± 0.01 a	6.03 ± 0.01 b	38.02 ± 0.11 a	51.64 ± 0.08 a	49.45 ± 0.10 b	29.25 ± 0.09 a	3.35 ± 0.02 c
Control	2.54 ± 0.02 d	2.12 ± 0.02 d	2.84 ± 0.02 c	1.73 ± 0.02 c	2.49 ± 0.01 d	1.70 ± 0.01 c	4.97 ± 0.01 d	30.67 ± 0.11 c	41.48 ± 0.08 d	40.46 ± 0.10 c	24.11 ± 0.09 c	2.74 ± 0.02 d
Significance*												
Y	*F* = 37.459 *p* < 0.0001	*F* = 918.124 *p* < 0.0001	*F* = 6591.751 *p* < 0.0001	*F* = 66.831 *p* < 0.0001	*F* = 34,857.304 *p* < 0.0001	*F* = 16,217.777 *p* < 0.0001	*F* = 19,517.140 *p* < 0.0001	*F* = 34,813.595 *p* < 0.0001	*F* = 37,955.303 *p* < 0.0001	*F* = 16,279.686 *p* < 0.0001	*F* = 22,674.186 *p* < 0.0001	*F* = 13,935.061 *p* < 0.0001
T	*F* = 239.154 *p* < 0.0001	*F* = 1518.407 *p* < 0.0001	*F* = 851.581 *p* < 0.0001	*F* = 177.439 *p* < 0.0001	*F* = 30,717.978 *p* < 0.0001	*F* = 8919.112 *p* < 0.0001	*F* = 19,588.734 *p* < 0.0001	*F* = 18,479.020 *p* < 0.0001	*F* = 34,260.908 *p* < 0.0001	*F* = 13,643.893 *p* < 0.0001	*F* = 15,724.849 *p* < 0.0001	*F* = 932.425 *p* < 0.0001
F	*F* = 208.509 *p* < 0.0001	*F* = 259.247 *p* < 0.0001	*F* = 60.287 *p* < 0.0001	*F* = 40.109 *p* < 0.0001	*F* = 4866.854 *p* < 0.0001	*F* = 1028.847 *p* < 0.0001	*F* = 2230.825 *p* < 0.0001	*F* = 1411.921 *p* < 0.0001	*F* = 4313.655 *p* < 0.0001	*F* = 3005.856 *p* < 0.0001	*F* = 789.820 *p* < 0.0001	*F* = 5313.215 *p* < 0.0001
Y × T	*F* = 1165.609 *p* < 0.0001	*F* = 2068.336 *p* < 0.0001	*F* = 708.206 *p* < 0.0001	*F* = 706.918 *p* < 0.0001	*F* = 17,949.386 *p* < 0.0001	*F* = 7985.882 *p* < 0.0001	*F* = 10,819.313 *p* < 0.0001	*F* = 18,218.609 *p* < 0.0001	*F* = 21,446.376 *p* < 0.0001	*F* = 10,129.672 *p* < 0.0001	*F* = 4402.728 *p* < 0.0001	*F* = 9639.177 *p* < 0.0001
Y × F	*F* = 86.561 *p* < 0.0001	*F* = 138.409 *p* < 0.0001	*F* = 89.569 *p* < 0.0001	*F* = 118.883 *p* < 0.0001	*F* = 3404.332 *p* < 0.0001	*F* = 536.276 *p* < 0.0001	*F* = 722.765 *p* < 0.0001	*F* = 1253.016 *p* < 0.0001	*F* = 3077.413 *p* < 0.0001	*F* = 4246.566 *p* < 0.0001	*F* = 541.177 *p* < 0.0001	*F* = 3662.599 *p* < 0.0001
T × F	*F* = 129.410 *p* < 0.0001	*F* = 391.912 *p* < 0.0001	*F* = 69.403 *p* < 0.0001	*F* = 80.266 *p* < 0.0001	*F* = 8030.554 *p* < 0.0001	*F* = 1398.358 *p* < 0.0001	*F* = 4448.376 *p* < 0.0001	*F* = 1581.655 *p* < 0.0001	*F* = 9223.965 *p* < 0.0001	*F* = 4288.816 *p* < 0.0001	*F* = 3222.030 *p* < 0.0001	*F* = 503.987 *p* < 0.0001
Y × T × F	*F* = 421.547 *p* < 0.0001	*F* = 355.426 *p* < 0.0001	*F* = 61.217 *p* < 0.0001	*F* = 244.063 *p* < 0.0001	*F* = 7482.558 *p* < 0.0001	*F* = 881.157 *p* < 0.0001	*F* = 2738.568 *p* < 0.0001	*F* = 2011.816 *p* < 0.0001	*F* = 7884.657 *p* < 0.0001	*F* = 3129.685 *p* < 0.0001	*F* = 1873.146 *p* < 0.0001	*F* = 2176.353 *p* < 0.0001

*Note:* ^X^Mean separation in years; ^Y^Mean separation in tillage; ^Z^Mean separation in fertilizer, Y × T, Y × F, T × F, Y × T × F, interactions; For a given factor (different letters within a column represent significant differences (Duncan's test, *, Significant at *p*‐value < 0.0001)). Data is expressed as a means of the data ± standard error (SE).

**FIGURE 1:**
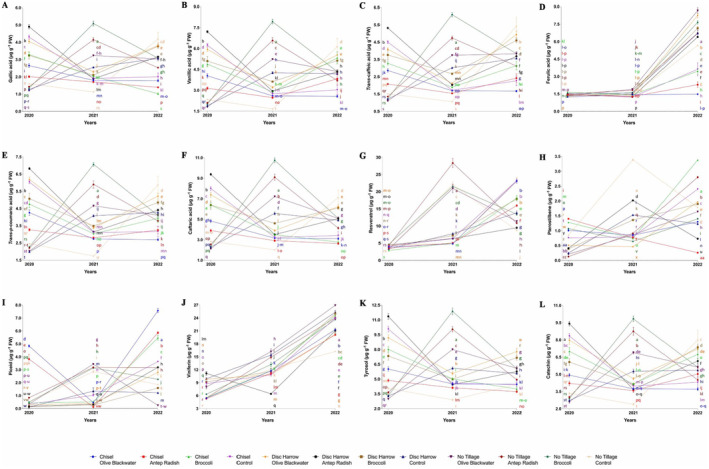
Effect of soil management on phenolic compounds in ‘Royal’ grape cultivar depending on fertilizer application and years of research.

Overall, the study revealed complex interactions between tillage methods, fertilizer applications, and years on phenolic compound accumulation in ‘Royal’ grapes. No tillage and broccoli fertilizer often yielded high levels of various compounds, particularly in 2021. Disc harrow and chisel tillage methods also showed effectiveness for certain compounds. The year 2022 generally saw increases in several phenolic compounds across treatments. Olive blackwater and Antep radish fertilizers also demonstrated positive effects on some compounds in specific years and tillage combinations. For gallic acid (Figure 1A), highest level in 2021 with disc harrow and broccoli fertilizer (5.08 μg g^−1^ FW); lowest in 2022 with chisel tillage and broccoli fertilizer (1.02 μg g^−1^ FW). Considering vanillic acid (Figure 1B), peaked in 2021 with no tillage and broccoli fertilizer (7.93 μg g^−1^ FW); lowest in 2021 with no tillage and control (1.66 μg g^−1^ FW). For *trans*‐caffeic acid (Figure 1C), highest in 2021 with no tillage and broccoli fertilizer (6.09 μg g^−1^ FW); lowest in 2021 with no tillage control (1.06 μg g^−1^ FW). Regarding ferulic acid (Figure 1D), peaked in 2022 with no tillage and olive blackwater (8.67 μg g^−1^ FW); lowest levels in 2020 and 2021. For *trans*‐*p*‐coumaric acid (Figure 1E), highest in 2021 with no tillage and broccoli (7.06 μg g^−1^ FW); lowest with disc harrow control in 2020. Given caftaric acid (Figure 1F), peaked in 2021 with no tillage and broccoli (10.75 μg g^−1^ FW); high levels also in 2020 with chisel tillage control (8.00 μg g^−1^ FW) and disc harrow with Antep radish (9.38 μg g^−1^ FW). For resveratrol (Figure 1G), highest in 2021 with no tillage and Antep radish (28.35 μg g^−1^ FW); significant increases in 2022 across treatments. Considering pterostilbene (Figure 1H), peaked in 2022 with chisel tillage and broccoli (3.38 μg g^−1^ FW); high levels also in 2021 with no tillage control (3.39 μg g^−1^ FW). For piceid (Figure 1I), highest in 2022 with chisel tillage and olive blackwater (7.57 μg g^−1^ FW); followed by chisel tillage with Antep radish (5.86 μg g^−1^ FW) and broccoli (5.47 μg g^−1^ FW). Regarding viniferin (Figure 1J), peaked in 2022 with no tillage and olive blackwater (26.94 μg g^−1^ FW); lowest in 2020 with chisel tillage using various fertilizers (5.29–5.52 μg g^−1^ FW). For tyrosol (Figure 1K), highest in 2021 with no tillage and broccoli (11.87 μg g^−1^ FW) and in 2020 with disc harrow and Antep radish (11.36 μg g^−1^ FW); lowest in 2022 with chisel tillage and Antep radish (3.73 μg g^−1^ FW). Lastly, for catechin (Figure 1L), peaked in 2021 with no tillage and broccoli (9.84 μg g^−1^ FW) and in 2020 with disc harrow and Antep radish (9.42 μg g^−1^ FW); lowest in 2021 with no tillage control (2.39 μg g^−1^ FW).

**FIGURE 2:**
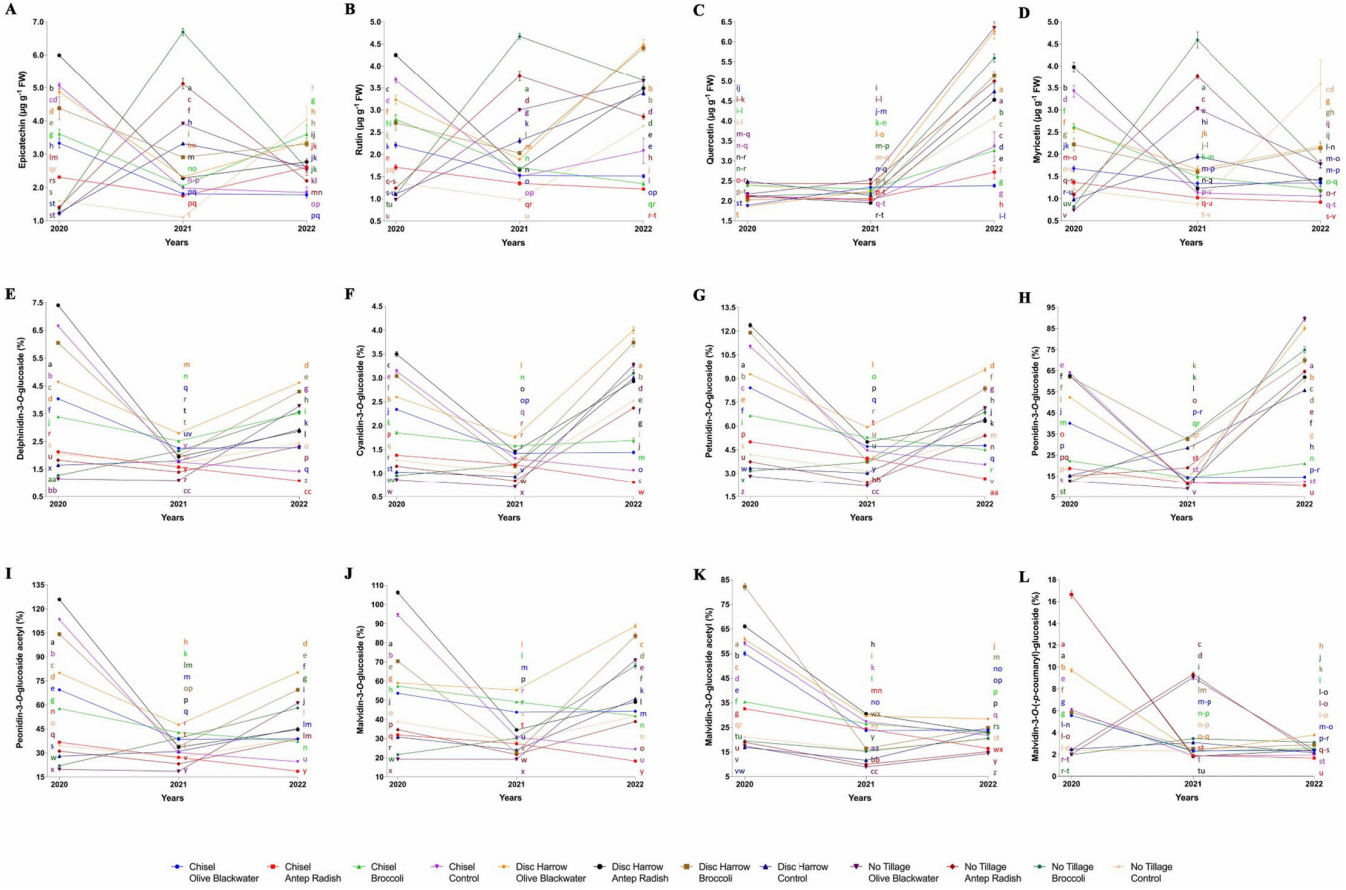
Effect of soil management on phenolic compounds in ‘Royal’ grape cultivar depending on fertilizer application and years of research.

In the study, there were significant differences in phenolic compound concentrations depending on different soil tillage methods and fertilizer applications between 2020 and 2022. Epicatechin content varied significantly across treatments, with the highest value of 6.69 μg g^−1^ FW observed under no tillage using broccoli fertilizer in 2021, while the lowest value of 1.10 μg g^−1^ FW was recorded with no tillage and control in the same year (Figure 2A). Rutin levels peaked at 4.67 μg g^−1^ FW under no tillage with broccoli fertilizer in 2021, contrasting with the lowest level of 0.98 μg g^−1^ FW found under no tillage with control treatment (Figure 2B). The study revealed that quercetin concentrations were most pronounced in 2022, reaching a maximum of 6.35 μg g^−1^ FW under no tillage with olive blackwater fertilizer (Figure 2C). Myricetin content exhibited its highest level of 4.59 μg g^−1^ FW in 2021 under no tillage using broccoli fertilizer, demonstrating the potential influence of specific fertilizer treatments on phenolic compound production (Figure 2D). Anthocyanin profiles showed distinct patterns across treatments. Delphinidin‐3‐*O*‐glucoside content reached its peak at 7.40% under disc harrow with Antep radish in 2020 (Figure 2E). Cyanidin‐3‐*O*‐glucoside levels were highest at 4.00% in 2022 with disc harrow using olive blackwater (Figure 2F), while petunidin‐3‐*O*‐glucoside demonstrated a maximum value of 12.37% under disc harrow with Antep radish in 2020 (Figure 2G). A notable finding was the dramatic increase in peonidin‐3‐*O*‐glucoside levels by 2022, reaching 89.50% under no tillage with olive blackwater treatment (Figure 2H). Peonidin‐3‐*O*‐glucoside acetyl content showed the highest concentration of 125.94% under disc harrow with Antep radish in 2020, indicating a strong interaction between tillage method and fertilizer type (Figure 2I). Malvidin compounds also exhibited significant variations across treatments and years. Malvidin‐3‐*O*‐glucoside percentage peaked at 106.25% under disc harrow with Antep radish in 2020 (Figure 2J), while malvidin‐3‐*O*‐glucoside acetyl content reached its maximum of 82.18% in 2020 with disc harrow using broccoli fertilizer (Figure 2K). Malvidin‐3‐*O*‐(‐*p*‐coumaryl)‐glucoside content was highest at 16.64% in 2020 under both chisel and disc harrow treatments using Antep radish (Figure 2L).

**FIGURE 3:**
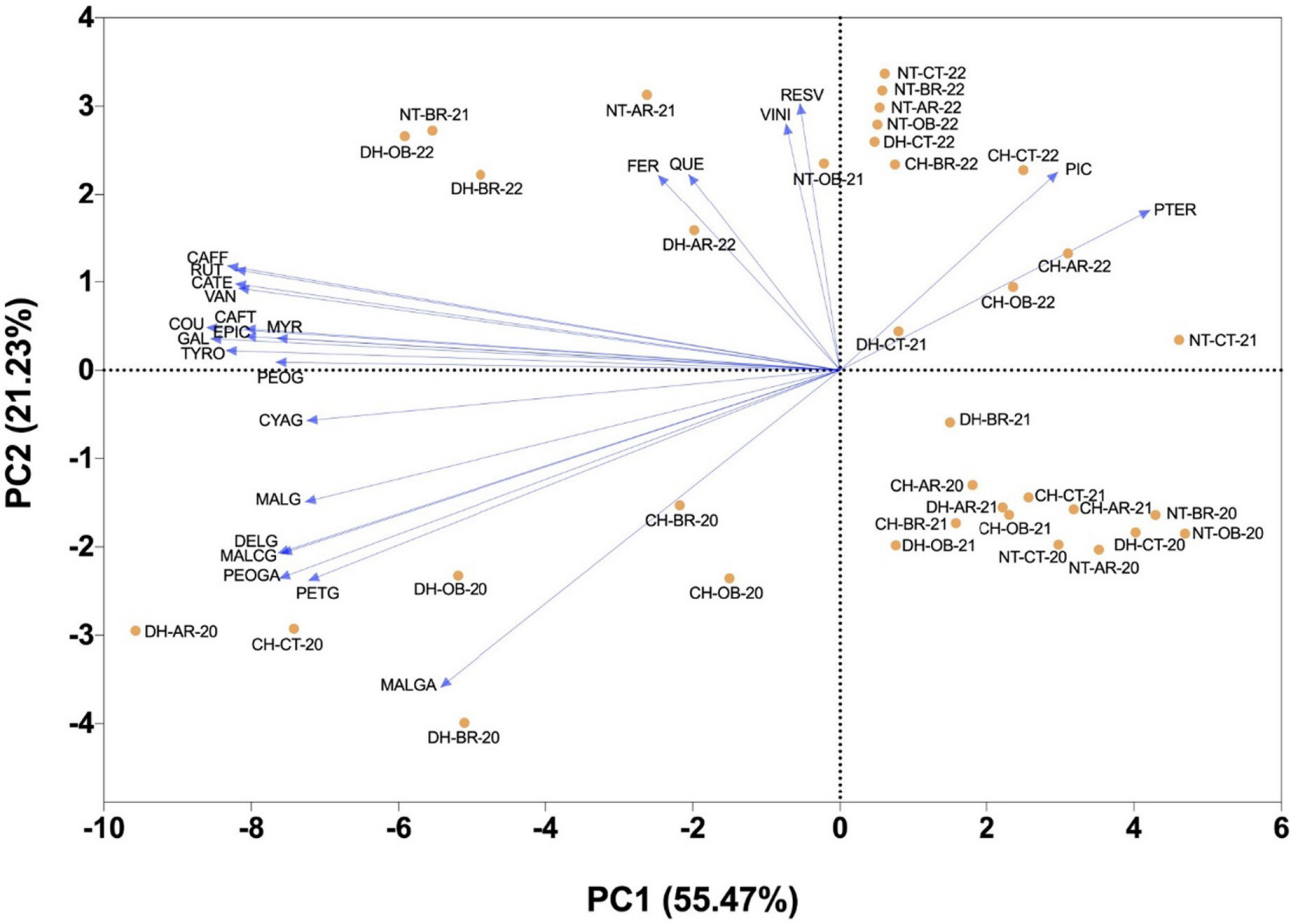
Principal component analysis (PCA) showing relative concentrations of phenolic compounds in ‘Royal’ grape variety depending on year, fertilizer application and soil management. PCA represents: PTER: Pterostilbene; PIC: Piceid; VINI: Viniferin; RESV: Resveratrol; QUE: Quercetin; FER: Ferulic acid; MALGA: Malvidin‐3‐*O*‐glucoside acetyl; PEOG: Peonidin‐3‐*O*‐glucoside; CYAG: Cyanidin‐3‐*O*‐glucoside; MALG: Malvidin‐3‐*O*‐glucoside; PEOGA: Peonidin‐3‐*O*‐glucoside acetyl; DELG: Delphinidin‐3‐*O*‐glucoside; MALCG: Malvidin‐3‐*O*‐(‐*p*‐coumaryl)‐glucoside; PETG: Petunidin‐3‐*O*‐glucoside; RUT: Rutin; EPIC: Epicatechin; CAFF: *Trans*‐caffeic acid; COU: *Trans*‐*p*‐coumaric acid; CATE: Catechin; VAN: Vanillic acid; GAL: Gallic acid; MYR: Myricetin; TYRO: Tyrosol; CAFT: Caftaric acid. For 2020, 2021 and 2022; Chisel Olive Blackwater (CH‐OB‐20), (CH‐OB‐21), (CH‐OB‐22), Chisel Antep Radish (CH‐AR‐20), (CH‐AR‐21), (CH‐AR‐22), Chisel Broccoli (CH‐BR‐20), (CH‐BR‐21), (CH‐BR‐22), Chisel Control (CH‐CT‐20), (CH‐CT‐21), (CH‐CT‐22), Disc Harrow Olive Blackwater (DH‐OB‐20), (DH‐OB‐21), (DH‐OB‐22), Disc Harrow Antep Radish (DH‐AR‐20), (DH‐AR‐21), (DH‐AR‐22), Disc Harrow Broccoli (DH‐BR‐20), (DH‐BR‐21), (DH‐BR‐22), Disc Harrow Control (DH‐CT‐20), (DH‐CT‐21), (DH‐CT‐22), No Tillage Olive Blackwater (NT‐OB‐20), (NT‐OB‐21), (NT‐OB‐22), No Tillage Antep Radish (NT‐AR‐20), (NT‐AR‐21), (NT‐AR‐22), No Tillage Broccoli (NT‐BR‐20), (NT‐BR‐21), (NT‐BR‐22), and No Tillage Control (NT‐CT‐20), (NT‐CT‐21) (NT‐CT‐22), respectively.

**FIGURE 4:**
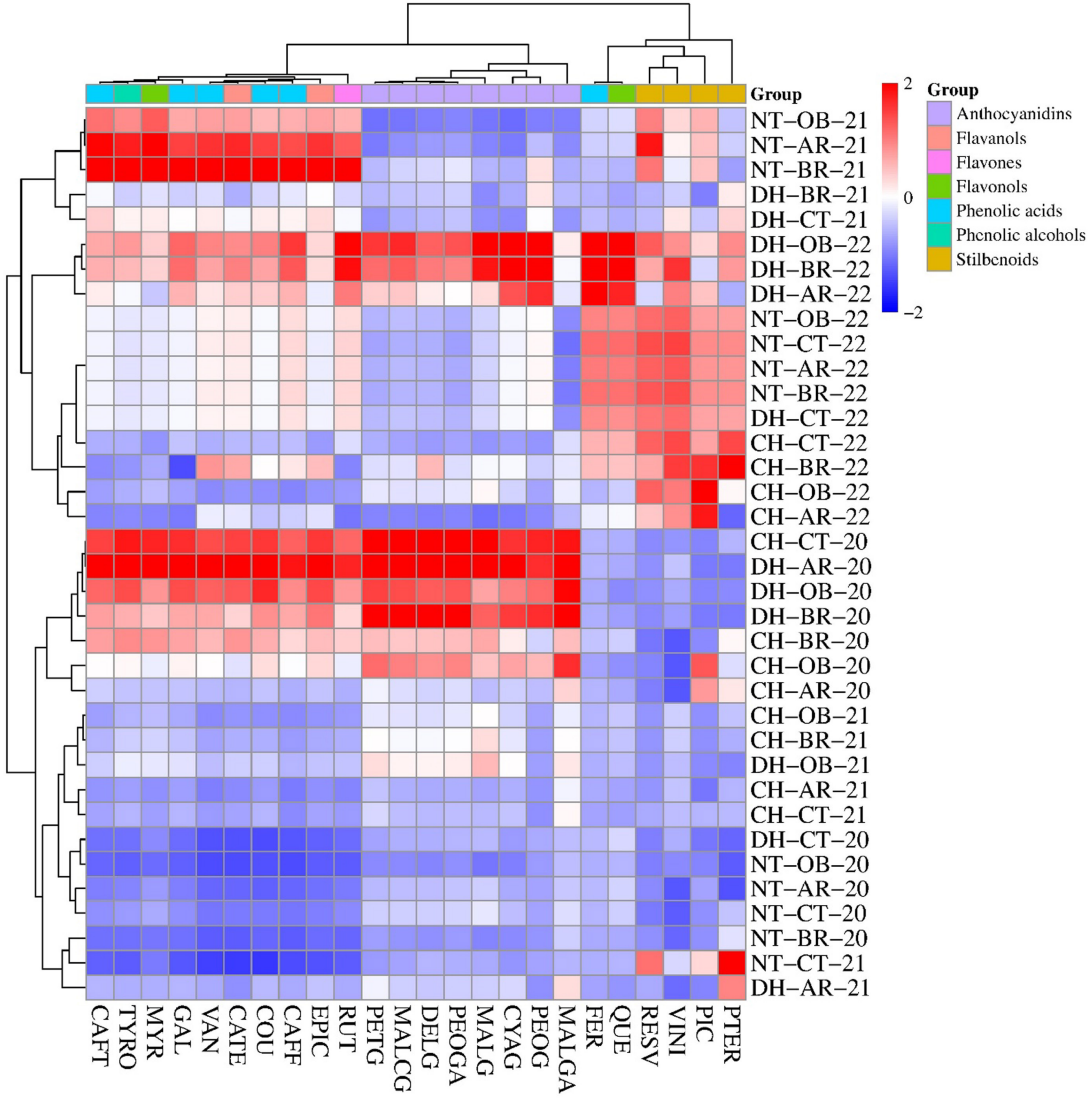
Hierarchical clustering heatmap analysis showing relative concentrations of phenolic compounds in ‘Royal’ grape variety depending on year, fertilizer application and soil management. Heatmap represents: PTER: Pterostilbene; PIC: Piceid; VINI: Viniferin; RESV: Resveratrol; QUE: Quercetin; FER: Ferulic acid; MALGA: Malvidin‐3‐*O*‐glucoside acetyl; PEOG: Peonidin‐3‐*O*‐glucoside; CYAG: Cyanidin‐3‐*O*‐glucoside; MALG: Malvidin‐3‐*O*‐glucoside; PEOGA: Peonidin‐3‐*O*‐glucoside acetyl; DELG: Delphinidin‐3‐*O*‐glucoside; MALCG: Malvidin‐3‐*O*‐(‐*p*‐coumaryl)‐glucoside; PETG: Petunidin‐3‐*O*‐glucoside; RUT: Rutin; EPIC: Epicatechin; CAFF: *Trans*‐caffeic acid; COU: *Trans*‐*p*‐coumaric acid; CATE: Catechin; VAN: Vanillic acid; GAL: Gallic acid; MYR: Myricetin; TYRO: Tyrosol; CAFT: Caftaric acid. For 2020, 2021 and 2022; Chisel Olive Blackwater (CH‐OB‐20), (CH‐OB‐21), (CH‐OB‐22), Chisel Antep Radish (CH‐AR‐20), (CH‐AR‐21), (CH‐AR‐22), Chisel Broccoli (CH‐BR‐20), (CH‐BR‐21), (CH‐BR‐22), Chisel Control (CH‐CT‐20), (CH‐CT‐21), (CH‐CT‐22), Disc Harrow Olive Blackwater (DH‐OB‐20), (DH‐OB‐21), (DH‐OB‐22), Disc Harrow Antep Radish (DH‐AR‐20), (DH‐AR‐21), (DH‐AR‐22), Disc Harrow Broccoli (DH‐BR‐20), (DH‐BR‐21), (DH‐BR‐22), Disc Harrow Control (DH‐CT‐20), (DH‐CT‐21), (DH‐CT‐22), No Tillage Olive Blackwater (NT‐OB‐20), (NT‐OB‐21), (NT‐OB‐22), No Tillage Antep Radish (NT‐AR‐20), (NT‐AR‐21), (NT‐AR‐22), No Tillage Broccoli (NT‐BR‐20), (NT‐BR‐21), (NT‐BR‐22), and No Tillage Control (NT‐CT‐20), (NT‐CT‐21) (NT‐CT‐22), respectively.

Considering principal component analysis (Figure 3), the first principal component (PC1), accounting for 55.47% of the variation, separated the treatments primarily along the horizontal axis, whereas the second principal component (PC2) explained an additional 21.23% of the variation, differentiating treatments along the vertical axis. Certain treatments, such as disc harrow with olive blackwater in 2022 and no tillage with olive blackwater in 2022, were positioned further along PC. This suggested a higher relative concentration of certain phenolic compounds like resveratrol and viniferin compared to other treatments. The grouping of chisel treatments from 2022 (chisel with Antep radish, chisel with broccoli, chisel with olive blackwater) near the top of PC2 indicated these combinations were associated with increased levels of another phenolic compound, pterostilbene, as indicated by the vector's direction. The control treatments under no tillage across all years (no tillage with control in 2020, no tillage with control in 2021, no tillage with control in 2022) were more closely associated with the baseline. Treatments like disc harrow with control in 2020 and chisel with control in 2020 have negative values on both principal components. Considering hierarchical clustering heatmap, the color gradient (from blue to red) indicated the scaled concentration levels of each compound, with red denoting higher concentrations and blue representing lower concentrations (Figure 4). The heatmap analysis has essentially classified the examined features into two main clusters. The first main cluster was further divided into two sub‐clusters. In the first sub‐cluster, phenolic acid group features (caftaric acid, gallic acid, vanillic acid, *trans*‐*p*‐coumaric acid, and *trans*‐caffeic acid), along with tyrosol, myricetin, catechin, epicatechin, and rutin, were predominantly found, whereas the second sub‐cluster comprised the anthocyanidin group including petunidin‐3‐*O*‐glucoside, malvidin‐3‐*O*‐(‐*p*‐coumaryl)‐glucoside, delphinidin‐3‐*O*‐glucoside, peonidin‐3‐*O*‐glucoside acetyl, malvidin‐3‐*O*‐glucoside, cyanidin‐3‐*O*‐glucoside, peonidin‐3‐*O*‐glucoside, and malvidin‐3‐*O*‐glucoside acetyl. The second main cluster was also divided into two sub‐clusters; the first sub‐cluster contained ferulic acid and quercetin features, along with stilbene group constituents resveratrol, viniferin, and piceid, while the second sub‐cluster uniquely hosted the pterostilbene feature, showing a distinct branching. The heatmap has also fundamentally categorized the applications involving different fertilization and soil tillage over the years into two main clusters. The first main cluster was divided into two sub‐clusters. The first sub‐cluster included applications with generally lower values in anthocyanidins and features such as caftaric acid, tyrosol, myricetin, gallic acid, vanillic acid, catechin, *trans*‐*p*‐coumaric acid, *trans*‐caffeic acid, epicatechin, and rutin, namely dics harrow with Antep radish in 2021, no tillage with control in 2021, no tillage with broccoli in 2020, no tillage with control in 2020, no tillage with Antep radish in 2020, no tillage with olive blackwater in 2020, disc harrow with control in 2020, chisel with control in 2021, chisel with Antep radish in 2021, disc harrow with olive blackwater in 2021, chisel with broccoli in 2021, and chisel with olive blackwater in 2021, while the second sub‐cluster, possessing higher averages in the same features, included chisel with Antep radish in 2020, chisel with olive blackwater in 2020, chisel with broccoli in 2020, disc harrow with broccoli in 2020, disc harrow with olive blackwater in 2020, disc harrow with Antep radish in 2020, and chisel with control in 2020 applications. Significant positive correlations were found between anthocyanidins and features such as caftaric acid, tyrosol, myricetin, gallic acid, vanillic acid, catechin, *trans*‐*p*‐coumaric acid, *trans*‐caffeic acid, epicatechin, and rutin in the second sub‐cluster, whereas these features showed strong negative correlations with resveratrol, viniferin, ferulic acid, and quercetin features. The second main cluster primarily consisted of applications from the year 2022 with higher averages in viniferin, ferulic acid, and quercetin features, forming the first sub‐cluster, including chisel with Antep radish in 2022, chisel with olive blackwater in 2022, chisel with broccoli in 2022, chisel with control in 2022, disc harrow with control in 2022, no tillage with broccoli in 2022, no tillage with Antep radish in 2022, no tillage with control in 2022, no tillage with olive blackwater in 2022, disc harrow with Antep radish in 2022, disc harrow with broccoli in 2022, and disc harrow with olive blackwater in 2022, while applications with lower averages in the same features, disc harrow with control in 2021, disc harrow with broccoli in 2021, no tillage with broccoli in 2021, no tillage with Antep radish in 2021, and no tillage with olive blackwater in 2021, formed the second sub‐cluster. Strong positive correlations were detected between caftaric acid, tyrosol, myricetin, gallic acid, vanillic acid, catechin, *trans*‐*p*‐coumaric acid, *trans*‐caffeic acid, epicatechin, and rutin features and resveratrol and piceid in the second sub‐cluster, whereas these features exhibited significant negative correlations with anthocyanidins, ferulic acid, quercetin, and pterostilbene features. When considering all features collectively, the applications most effective in the accumulation of phenolic compounds, in order, were disc harrow with olive blackwater in 2022, disc harrow with broccoli in 2022, and disc harrow with Antep radish in 2022.

## 4 Discussion

This study investigated the effects of tillage and organic fertilizer treatments on phenolic compounds and anthocyanins in the Royal grape cultivar. The primary focus was on understanding how soil tillage methods, integrated with organic fertilization, influence these key compounds in grapes, which are critical to both grape quality and overall vine productivity. The study specifically explored the impact of different tillage strategies, disk harrow, chisel tillage, and no tillage, alongside organic fertilization applications, on the soil water balance and vineyard microclimate, which are vital for vine water and mineral uptake. Our findings revealed that both tillage treatments and organic fertilizer applications significantly affected the levels of phenolic compounds and anthocyanins in the Royal grape cultivar, as evidenced by the data presented in Table 1, Figures 1 and 2, and the Data S1. The results corroborate previous studies by Bahar and Yasasin (2010), Gambacorta et al. (2011), and Buesa et al. (2021), which have shown that phenolic compounds and anthocyanin levels in grapes are highly sensitive to tillage and fertilization practices. Among the tillage treatments, disc harrow emerged as the most effective method for enhancing the levels of various phenolic compounds and anthocyanins in Royal grape berries. This treatment led to the highest concentrations of several phenolic acids, including gallic, vanillic, trans‐caffeic, *trans*‐*p*‐coumaric, and caftaric acids. Additionally, disc harrowing resulted in peak levels of stilbenes such as viniferin and tyrosol, as well as flavanols like catechin and epicatechin, and the flavonol rutin. Regarding anthocyanin profiles, the disc harrow treatment was associated with the maximal accumulation of anthocyanins such as peonidin‐3‐*O*‐glucoside, delphinidin‐3‐*O*‐glucoside, petunidin‐3‐*O*‐glucoside, cyanidin‐3‐*O*‐glucoside, peonidin‐3‐*O*‐glucoside acetyl, malvidin‐3‐*O*‐glucoside, malvidin‐3‐*O*‐glucoside acetyl, and malvidin‐3‐*O*‐(‐*p*‐coumaroyl)‐glucoside in the grape berries. These findings, to the best of our knowledge, are unprecedented, as no previous studies have reported such high levels of these phenolic compounds in grapes under disc harrow treatment compared to other tillage methods or no tillage. The enhanced phenolic profiles observed with disc harrow treatment can be attributed to two primary factors. First, the disc harrow application likely improved soil aeration by effectively breaking up soil clods, which in turn enhanced water retention capacity. Improved aeration and water availability are crucial for reducing edaphic stress in vines by promoting root development and facilitating the uptake of minerals, potentially stimulating the biosynthesis of phenolic compounds and anthocyanins through the activation of defense mechanisms in the berries. Second, the disc harrow treatment may have increased the organic matter content in the soil by incorporating surface‐dwelling weeds and vine residues. Organic matter improves soil structure and supports microbial activity, which may have further contributed to vine growth and the production of phenolic compounds and anthocyanins. These hypotheses align with recent research, which emphasizes the role of soil water supply, temperature, and mineral availability as key determinants of vine growth and grape quality (Delpuech and Metay 2018; Van Leeuwen, Roby, and De Resseguier 2018; Martínez‐Fernández, González‐Zamora, and Almendra‐Martín 2021). While this study did not explore the intricate relationships between climate, soil characteristics, soil water balance, and mineral absorption, it clearly demonstrated the influence of specific tillage management practices, particularly disc harrow, on the accumulation of phenolic compounds and anthocyanins in Royal grape berries. Our results underscore the importance of integrated soil and organic fertilization management in enhancing vineyard productivity and grape quality, particularly through the modulation of key secondary metabolites essential for grape and wine quality.

In our study, the concentrations of phenolic compounds and anthocyanins in the Royal grape cultivar exhibited variability depending on the year (Table 1, Figures 1 and 2 and Data S1). The observed variations in these components' years are thought to be largely associated with the climatic conditions specific to the years in which the studies were conducted. It has, indeed, been reported that climatic variables, particularly rainfall amount and distribution, soil moisture levels, as well as atmospheric temperature, can induce water stress in vines, which may trigger an increase in secondary metabolites, namely phenolic compounds, and anthocyanins (Rienth et al. 2021; Shah et al. 2021). This phenomenon suggests that vines respond to stress conditions by increasing the production of phenolic compounds and anthocyanins as part of their adaptation mechanisms, providing protection against oxidative damage that increases under stress. In this context, it is recognized that changes in atmospheric temperature and precipitation patterns can directly and indirectly affect the phenolic profile of grapevines. These alterations influence the vine's water and mineral uptake capacity, photosynthetic activity, and overall metabolic responses, thereby impacting the biosynthesis pathways of secondary metabolites like phenolic compounds and anthocyanins. Understanding the effects of climatic conditions on the accumulation of these compounds can contribute to a better understanding of how vines adapt to environmental stressors, ultimately influencing their biosynthesis. This perspective is pivotal for optimizing agricultural management strategies and viticultural practices. Our study revealed significant variability in the concentrations of phenolic compounds and anthocyanins across different years, tillage methods, and fertilizer treatments, including organic options such as broccoli fertilizer, olive blackwater, and Antep radish. These fluctuations indicate the complex interactions between tillage, fertilizer treatments, and their combined impact on vine physiology and berry composition. Among the organic fertilizers, the broccoli‐based fertilizer emerged as particularly effective in enhancing the accumulation of several phenolic acids (vanillic, gallic, *trans*‐caffeic, ferulic, *trans‐p*‐coumaric, caftaric), stilbenes (viniferin, tyrosol), flavanols (catechin, epicatechin), and the flavonols (rutin) in grape berries. Additionally, it led to higher levels of anthocyanins such as cyanidin‐3‐*O*‐glucoside, malvidin‐3‐*O*‐glucoside acetyl, peonidin‐3‐*O*‐glucoside, delphinidin‐3‐*O*‐glucoside, and peonidin‐3‐*O*‐glucoside acetyl. These findings suggest that the application of broccoli fertilizer, being an organic amendment, may improve soil fertility, water‐holding capacity, and microbial activity, thereby promoting vine growth and the production of secondary metabolites. In addition, a significant increase in some phenolics occurred after the application of broccoli fertilizer, indicating that broccoli fertilizer decomposes in the soil over time and turns into organic matter. This can be explained by the fact that phenolics are involved in biosynthetic pathways that lead to the formation of humic substances as soon as they are broken down and released (Stevenson 1994; Santos et al. 2018). Previous studies have demonstrated that the utilization of organic fertilizers enhances the biosynthesis of phenolic compounds and anthocyanins in various crops (Malusà et al. 2002; Da Silva et al. 2024). The advantages of organic fertilizers arise from their ability to release essential minerals gradually, improve soil physical properties, and enhance the availability of micronutrients crucial for plant growth and the development of secondary metabolites (Dhaliwal et al. 2019; Bamdad et al. 2022).

In our study, the application of olive blackwater as a fertilizer demonstrated promising results, significantly enhancing the levels of petunidin‐3‐*O*‐glucoside, resveratrol, malvidin‐3‐*O*‐glucoside, and quercetin in the Royal grape cultivar. This effect is likely due to the presence of organic matter and bioactive compounds in olive blackwater, which may stimulate the biosynthesis of these specific phenolic compounds. Previous research has shown that olive mill waste can enhance phenolic production in various crops (Santos et al. 2018; Tapia‐Quirós et al. 2022). In contrast, the Antep radish fertilizer generally resulted in moderate to low levels of most phenolic compounds and anthocyanins, except for notable increases in piceid and malvidin‐3‐*O*‐(‐*p*‐coumaroyl)‐glucoside. Interestingly, in 2021, the combination of no tillage practices with Antep radish fertilizer led to a substantial increase in resveratrol content (28.35 μg g^−1^ FW), suggesting that specific agricultural practices can be adapted to enhance the health‐promoting properties of grapes. Additionally, the highest concentrations of tyrosol and catechin observed in 2020 with the disc harrow and Antep radish fertilizer combination (11.36 μg g^−1^ FW and 9.42 μg g^−1^ FW, respectively) further illustrate the synergistic effects of combining targeted fertilization with specific tillage methods to boost the accumulation of phenolic alcohols and flavonoids. The variability in phenolic response to different treatments could be linked to the unique mineral composition and properties of the Antep radish fertilizer, which may selectively influence the biosynthesis of certain compounds. Organic fertilizers, including Antep radish, are known to improve soil health by enhancing soil structure, increasing microbial activity, and providing a slow release of essential minerals, all of which are critical for the biosynthesis of phenolic compounds and anthocyanins in grapevines (Meissner et al. 2019). For example, nitrogen, a key component of amino acids, is crucial for the synthesis of phenylalanine, a precursor in the biosynthetic pathways for phenolics and anthocyanins (Cheng et al. 2020). The richer and more diverse microbial ecosystem promoted by organic fertilizers can enhance vine health and stress response mechanisms (Cataldo, Fucile, and Mattii 2021). Enhanced microbial activity may improve mineral uptake efficiency and stimulate plant defense mechanisms, leading to increased synthesis of secondary metabolites, including phenolics and anthocyanins, in response to biotic stress. Additionally, organic matter from fertilizers may have improved soil structure and water‐holding capacity, potentially mitigating water stress during dry conditions and indirectly promoting the accumulation of phenolic compounds and anthocyanins in grape berries.

The analysis of PCA and hierarchical clustering heatmap data, as presented in Figures 3 and 4 respectively, indicated profound insights into the relationship between tillage and fertilization and the phenolic compound profile of berries. Our PCA results indicated a significant variance in phenolic compound concentrations based on tillage and fertilization practices, with the first two principal components (PC1 and PC2) explaining over 76% of the variation. The positioning of treatments such as disc harrow with olive blackwater in 2022 and no tillage with olive blackwater in 2022 further along PC1 suggests that these practices are particularly effective in enhancing concentrations of resveratrol and viniferin. Minimal tillage practices, such as no tillage, increase plant stress due to less disturbance, leading to higher concentrations of stress‐induced phenolic compounds like resveratrol and viniferin. The association of chisel treatments from 2022 (e.g., chisel with Antep radish, chisel with broccoli, chisel with olive blackwater) with increased levels of pterostilbene near the top of PC2 supports the notion that specific mechanical soil management practices can influence the accumulation of particular phenolic compounds. The observed patterns in phenolic compound accumulation are hypothesized to be driven by the effects of soil management and fertilization practices on plant stress levels and mineral availability, influencing secondary metabolite synthesis. As proposed by Kaya, Ates, et al. (2024) and Kaya, Yilmaz, et al. (2024), chisel tillage‐induced soil disruption might create conditions around plant roots conducive to the production of specific secondary compounds. Through heatmap analysis, compounds are categorized into groups and sub‐groups based on agricultural techniques, illustrating the intricate relationship between soil management, fertilization strategies, and their impact on plant secondary metabolite levels. These patterns and connections suggest that certain farming methods are particularly effective in enhancing the production of specific phenolic compounds. These findings corroborate previous research by Dhaliwal et al. (2019) and Bamdad et al. (2022), which indicated that organic fertilization and conservation tillage can elevate secondary metabolite levels in grapes.

## 5 Conclusion

Based on the findings of our 3‐year investigation, we observed that the levels of phenolic and anthocyanin compounds in grape berries varied significantly from year to year. These variations highlighted the significant influence of environmental and climatic factors on the compositional profiles. Specifically, the notable rise in phenolic concentrations linked to the disc harrow method underscored the crucial role of precise tillage in enhancing the accumulation of these essential phytochemicals. Furthermore, the marked increase in specific phenolics, like gallic acid, and anthocyanins, due to the combined use of disc harrow tillage and broccoli‐based organic fertilizer, suggested a synergistic effect between the enrichment of organic matter and the improvement of soil aeration. This synergy not only indicates the advantages of incorporating organic broccoli fertilizer for enhanced grape quality but also aligns with the principles of sustainable viticulture practices. The results support the implementation of certain agricultural practices, like the strategic combination of organic broccoli fertilizer and the disc harrow method, to notably enhance grape quality, thereby fostering a sustainable and quality‐focused approach to viticulture. However, the long‐term impacts of these agricultural interventions on soil health and vine productivity, along with their ability to withstand the variations in phenolic compounds induced by climate change, require additional research. To sum up, the detailed insights obtained from our study offer crucial guidance for viticulturists on optimizing the phenolic and anthocyanin profiles of grapes, enhancing both the berry quality and its health benefits, thus contributing to the advancement of viticultural science and practice.

## Author Contributions

**Ozkan Kaya:** conceptualization (equal), data curation (equal), formal analysis (equal), funding acquisition (equal), investigation (equal), methodology (equal), project administration (equal), resources (equal), software (equal), supervision (equal), validation (equal), visualization (equal), writing – original draft (equal), writing – review and editing (equal). **Fadime Ates:** conceptualization (equal), data curation (equal), formal analysis (equal), resources (equal), validation (equal). **Selda Daler:** conceptualization (equal), formal analysis (equal). **Sevil Canturk:** formal analysis (equal), resources (equal), software (equal). **Metin Turan:** investigation (equal), software (equal), visualization (equal). **Harlene Hatterman‐Valenti:** resources (equal), validation (equal), visualization (equal).

## Ethics Statement

The authors have nothing to report.

## Consent

The authors have nothing to report.

## Conflicts of Interest

The authors declare no conflicts of interest.

## Supporting information


Data S1.

## Data Availability

Requests for materials should be directed to O.K. and F.A.
