# Supplementary material for: Optimizing Grape Quality Through Tillage and Organic Fertilization: A Comprehensive Analysis of Phenolic and Anthocyanin Variability Over Three Years
Source: Food Sci Nutr. 2024 Oct 10;12(11):9428–42. doi: 10.1002/fsn3.4500 (PMC11606872; doi:10.1002/fsn3.4500)
Supplement: Supplementary file 1 — Data S1. [file FSN3-12-9428-s001.docx]

| **Supplementary Tables.** Phenolic compounds in 'Royal' grape varieties subject to different tillage and fertilizer treatments over three years  **Gallic acid (µg g^-1^ FW)** | | | | | |
| --- | --- | --- | --- | --- | --- |
| **Tillage** | **Fertilizer** | **Years** | | | ***Average of years*** |
|  |  | **2020** | **2021** | **2022** |  |
| **Chisel** | Olive Blackwater | 2.65±0.11 i^*^ | 1.80±0.05 mn | 1.78±0.07 m-o | *2.08±0.43 Cb*^**^ |
|  | Antep Radish | 2.01±0.06 kl | 1.70±0.04 no | 1.39±0.01 p | *1.70±0.27 Cc* |
|  | Broccoli | 3.29±0.13 f | 2.03±0.06 kl | 1.02±0.05 s | *2.11±0.99 Cb* |
|  | Control | 4.30±0.11 c | 1.91±0.06 k-m | 2.00±0.22 kl | *2.74±1.18 Aa* |
| **Disc Harrow** | Olive Blackwater | 4.05±0.10 d | 2.28±0.06 j | 3.81±0.14 e | *3.38±0.84 Aa* |
|  | Antep Radish | 4.90±0.12 b | 1.89±0.01 lm | 3.16±0.05 f-h | *3.32±1.31 Aa* |
|  | Broccoli | 3.24±0.21 fg | 2.10±0.01 k | 3.77±0.11 e | *3.04±0.75 Bb* |
|  | Control | 1.27±0.03 p-r | 2.55±0.04 i | 3.06±0.02 gh | *2.29±0.80 Bc* |
| **No Tillage** | Olive Blackwater | 1.20±0.03 q-s | 3.23±0.02 f-h | 3.04±0.11 h | *2.49±0.97 Bc* |
|  | Antep Radish | 1.43±0.04 p | 4.14±0.12 cd | 2.53±0.04 i | *2.70±1.19 Bb* |
|  | Broccoli | 1.34±0.04 pq | 5.08±0.14 a | 3.08±0.09 gh | *3.17±1.62 Aa* |
|  | Control | 1.60±0.04 o | 1.12±0.04 rs | 4.19±0.38 cd | *2.30±1.44 Bd* |
| ***Average of tillage methods*** | *Chisel* | *3.06±0.89 Ab*^***^ | *1.86±0.13 Bc* | *1.55±0.41 Cc* | *2.16±0.86 c* |
|  | *Disc Harrow* | *3.37±1.41 Ba* | *2.21±0.25 Cb* | *3.45±0.37 Aa* | *3.01±1.01 a* |
|  | *No Tillage* | *1.39±0.16 Cc* | *3.39±1.54 Aa* | *3.21±0.65 Bb* | *2.67±1.31 b* |
| ***Average of fertilizers*** | *Olive Blackwater* | *2.63±1.24 Bb* | *2.44±0.63 Cc* | *2.88±0.89 Ab* | *2.65±0.93 b* |
|  | *Antep Radish* | *2.78±1.61 Aa* | *2.58±1.18 Bb* | *2.36±0.78 Cd* | *2.57±1.20 c* |
|  | *Broccoli* | *2.63±0.97 Bb* | *3.07±1.51 Aa* | *2.62±1.24 Bc* | *2.77±1.23 a* |
|  | *Control* | *2.39±1.44 Bc* | *1.86±0.62 Cd* | *3.08±0.97 Aa* | *2.44±1.14 d* |
| **Vanillic acid (µg g^-1^ FW)** | | | | | |
| **Tillage** | **Fertilizer** | **Years** | | | ***Average of years*** |
|  |  | **2020** | **2021** | **2022** |  |
| **Chisel** | Olive Blackwater | 4.04±0.15 hi^*^ | 2.60±0.05 m-o | 2.57±0.08 m-o | *3.07±0.74 Cc*^**^ |
|  | Antep Radish | 3.15±0.09 k | 2.47±0.06 no | 3.83±0.40 ij | *3.15±0.62 Cc* |
|  | Broccoli | 4.88±0.19 g | 2.89±0.05 k-m | 5.33±0.43 e | *4.37±1.15 Ba* |
|  | Control | 6.24±0.12 d | 2.75±0.07 l-n | 3.03±0.33 kl | *4.01±1.69 Ab* |
| **Disc Harrow** | Olive Blackwater | 5.95±0.16 d | 3.21±0.06 k | 5.54±0.21 e | *4.90±1.28 Aa* |
|  | Antep Radish | 7.21±0.08 b | 2.94±0.06 kl | 4.33±0.08 h | *4.83±1.89 Aa* |
|  | Broccoli | 5.10±0.34 fg | 3.62±0.03 j | 5.13±0.15 fg | *4.62±0.77 Ab* |
|  | Control | 1.89±0.04 qr | 4.26±0.08 h | 4.18±0.06 h | *3.44±1.17 Bc* |
| **No Tillage** | Olive Blackwater | 1.80±0.04 qr | 5.23±0.04 e | 4.35±0.13 h | *3.79±1.54 Bc* |
|  | Antep Radish | 2.10±0.04 pq | 6.58±0.20 c | 3.71±0.07 j | *4.13±1.97 Bb* |
|  | Broccoli | 2.00±0.06 q | 7.93±0.16 a | 4.34±0.12 h | *4.76±2.59 Aa* |
|  | Control | 2.34±0.05 op | 1.66±0.04 r | 6.14±0.59 d | *3.38±2.11 Bd* |
| ***Average of tillage methods*** | *Chisel* | *4.58±1.20 Ab*^***^ | *2.68±0.17 Cc* | *3.69±1.13 Bb* | *3.65±1.22 c* |
|  | *Disc Harrow* | *5.04±2.06 Aa* | *3.51±0.52 Cb* | *4.79±0.60 Ba* | *4.45±1.41 a* |
|  | *No Tillage* | *2.06±0.21 Cc* | *5.35±2.44 Aa* | *4.63±0.98 Ba* | *4.01±2.06 b* |
| ***Average of fertilizers*** | *Olive Blackwater* | *3.93±1.80 Bb* | *3.68±1.19 Cc* | *4.15±1.30 Ac* | *3.92±1.41 c* |
|  | *Antep Radish* | *4.15±2.34 Aa* | *4.00±1.95 ABb* | *3.95±0.35 Bc* | *4.04±1.70 b* |
|  | *Broccoli* | *4.00±1.51 Bb* | *4.82±2.36 Aa* | *4.93±0.51 Aa* | *4.58±1.64 a* |
|  | *Control* | *3.49±2.07 Bc* | *2.89±1.13 Cd* | *4.45±1.40 Ab* | *3.61±1.66 d* |
| ^*^Different lowercase letters indicate differences according to the tillage × fertilizer × year interaction (P<0.05). ^**^Different lowercase letters indicate differences among fertilizer means within the same soil tillage method, whereas different uppercase letters indicate differences of the same fertilizer across different soil tillage methods (P<0.05). ^***^Different lowercase letters indicate differences among means in the same column, whereas different uppercase letters indicate differences among means in the same row (P<0.05).  Data are presented as mean + standard deviation (SD). | | | | | |
|  |  |  |  |  |  |
| ***Trans*-caffeic acid (µg g^-1^ FW)** | | | | | |
| **Tillage** | **Fertilizer** | **Years** | | | ***Average of years*** |
|  |  | **2020** | **2021** | **2022** |  |
| **Chisel** | Olive Blackwater | 2.86±0.10 jk^*^ | 1.69±0.03 op | 1.67±0.05 op | *2.07±0.59 Cb*^**^ |
|  | Antep Radish | 2.08±0.04 mn | 1.54±0.03 pq | 2.42±0.25 l | *2.01±0.40 Cb* |
|  | Broccoli | 3.28±0.10 h | 1.90±0.04 no | 3.14±0.24 hi | *2.77±0.67 Ba* |
|  | Control | 4.50±0.09 d | 1.72±0.03 op | 2.26±0.28 lm | *2.83±1.28 Aa* |
| **Disc Harrow** | Olive Blackwater | 4.09±0.08 e | 2.12±0.04 mn | 4.94±0.19 c | *3.72±1.25 Aa* |
|  | Antep Radish | 5.32±0.04 b | 2.03±0.06 mn | 3.68±0.04 fg | *3.68±1.43 Aa* |
|  | Broccoli | 3.76±0.29 fg | 2.67±0.02 k | 4.60±0.13 d | *3.68±0.85 Aa* |
|  | Control | 1.21±0.03 rs | 3.02±0.03 ij | 3.56±0.02 g | *2.60±1.07 Bb* |
| **No Tillage** | Olive Blackwater | 1.10±0.02 s | 3.74±0.02 fg | 3.83±0.12 f | *2.89±1.34 Bc* |
|  | Antep Radish | 1.35±0.03 qr | 4.74±0.11 cd | 3.12±0.02 hi | *3.07±1.47 Bb* |
|  | Broccoli | 1.23±0.03 rs | 6.09±0.12 a | 3.83±0.09 f | *3.72±2.11 Aa* |
|  | Control | 1.51±0.03 pq | 1.06±0.03 s | 5.43±0.52 b | *2.67±2.10 ABd* |
| ***Average of tillage methods*** | *Chisel* | *3.18±0.91 Ab*^***^ | *1.71±0.13 Cc* | *2.37±0.58 Bb* | *2.42±0.86 c* |
|  | *Disc Harrow* | *3.59±1.57 Ba* | *2.46±0.43 Cb* | *4.20±0.62 Aa* | *3.42±1.22 a* |
|  | *No Tillage* | *1.30±0.16 Cc* | *3.91±1.92 Ba* | *4.05±0.91 Aa* | *3.09±1.76 b* |
| ***Average of fertilizers*** | *Olive Blackwater* | *2.68±1.30 Bb* | *2.52±0.93 Cc* | *3.48±1.44 Ab* | *2.89±1.27 b* |
|  | *Antep Radish* | *2.92±1.83 Ba* | *2.77±1.49 Cb* | *3.07±0.56 Ac* | *2.92±1.35 b* |
|  | *Broccoli* | *2.76±1.17 Cb* | *3.55±1.93 Ba* | *3.86±0.65 Aa* | *3.39±1.39 a* |
|  | *Control* | *2.41±1.57 Bc* | *1.94±0.86 Cd* | *3.75±1.41 Aa* | *2.70±1.49 c* |
|  |  |  |  |  |  |
|  |  |  |  |  |  |
| **Ferulic acid (µg g^-1^ FW)** | | | | | |
| **Tillage** | **Fertilizer** | **Years** | | | ***Average of years*** |
|  |  | **2020** | **2021** | **2022** |  |
| **Chizel** | Olive Blackwater | 1.24±0.02 p^*^ | 1.45±0.03 l-p | 1.48±0.03 l-p | *1.39±0.12 Cc*^**^ |
|  | Antep Radish | 1.42±0.03 l-p | 1.27±0.03 op | 2.30±0.24 i | *1.66±0.50 Cb* |
|  | Broccoli | 1.65±0.02 kl | 1.42±0.03 l-p | 3.45±0.26 h | *2.17±0.97 Ca* |
|  | Control | 1.44±0.03 l-p | 1.24±0.03 p | 3.62±0.56 h | *2.10±1.18 Ca* |
| **Disc Harrow** | Olive Blackwater | 1.26±0.03 p | 1.40±0.02 l-p | 8.26±0.23 b | *3.64±3.47 Ba* |
|  | Antep Radish | 1.47±0.01 l-p | 1.25±0.04 p | 6.40±0.06 f | *3.04±2.52 Bc* |
|  | Broccoli | 1.39±0.03 l-p | 1.47±0.01 l-p | 7.15±0.15 d | *3.34±2.86 Bb* |
|  | Control | 1.54±0.03 l-o | 1.57±0.04 l-n | 6.71±0.28 e | *3.27±2.58 Ab* |
| **No Tillage** | Olive Blackwater | 1.34±0.03 m-p | 1.90±0.01 j | 8.67±0.25 a | *3.97±3.54 Aa* |
|  | Antep Radish | 1.51±0.03 l-p | 1.82±0.02 jk | 6.73±0.07 e | *3.35±2.54 Ac* |
|  | Broccoli | 1.32±0.03 n-p | 1.61±0.03 k-m | 7.66±0.15 c | *3.53±3.10 Ab* |
|  | Control | 1.48±0.03 l-p | 1.36±0.03 m-p | 5.68±0.05 g | *2.84±2.13 Bd* |
| ***Average of tillage methods*** | *Chisel* | *1.44±0.16 Ba*^***^ | *1.35±0.10 Bc* | *2.71±0.96 Ab* | *1.83±0.84 c* |
|  | *Disc Harrow* | *1.42±0.11 Bb* | *1.42±0.12 Bb* | *7.13±0.76 Aa* | *3.32±2.77 b* |
|  | *No Tillage* | *1.41±0.09 Cb* | *1.67±0.22 Ba* | *7.19±1.17 Aa* | *3.42±2.78 a* |
| ***Average of fertilizers*** | *Olive Blackwater* | *1.28±0.05 Cc* | *1.58±0.24 Ba* | *6.14±3.50 Aa* | *3.00±2.99 a* |
|  | *Antep Radish* | *1.47±0.04 Bab* | *1.45±0.28 Bc* | *5.14±2.14 Ab* | *2.69±2.14 b* |
|  | *Broccoli* | *1.46±0.15 Bb* | *1.50±0.09 Bb* | *6.09±2.00 Aa* | *3.01±2.48 a* |
|  | *Control* | *1.49±0.05 Ba* | *1.39±0.15 Bd* | *5.34±1.40 Ab* | *2.74±2.03 b* |
| ^*^Different lowercase letters indicate differences according to the tillage × fertilizer × year interaction (P<0.05). ^**^Different lowercase letters indicate differences among fertilizer means within the same soil tillage method, whereas different uppercase letters indicate differences of the same fertilizer across different soil tillage methods (P<0.05). ^***^Different lowercase letters indicate differences among means in the same column, whereas different uppercase letters indicate differences among means in the same row (P<0.05).  Data are presented as mean + standard deviation (SD). | | | | | |
|  |  |  |  |  |  |
| ***Trans-p*-coumaric acid (µg g^-1^ FW)** | | | | | |
| **Tillage** | **Fertilizer** | **Years** | | | ***Average of years*** |
|  |  | **2020** | **2021** | **2022** |  |
| **Chisel** | Olive Blackwater | 4.26±0.16 hi^*^ | 2.73±0.04 p | 2.69±0.07 pq | *3.23±0.78 Cc*^**^ |
|  | Antep Radish | 3.27±0.07 ln | 2.80±0.06 op | 3.25±0.21 ln | *3.11±0.26 Cc* |
|  | Broccoli | 4.76±0.21 g | 3.04±0.04 no | 3.93±0.25 jk | *3.91±0.76 Bb* |
|  | Control | 6.04±0.09 cd | 3.11±0.07 mn | 3.16±0.31 mn | *4.10±1.46 Aa* |
| **Disc Harrow** | Olive Blackwater | 6.19±0.14 c | 3.38±0.05 lm | 5.22±0.17 e | *4.93±1.24 Aa* |
|  | Antep Radish | 6.82±0.07 b | 3.15±0.01 mn | 4.41±0.06 h | *4.79±1.61 Ab* |
|  | Broccoli | 5.07±0.31 ef | 3.49±0.01 l | 4.85±0.12 fg | *4.47±0.76 Ac* |
|  | Control | 1.99±0.03 t | 4.08±0.06 ij | 4.27±0.03 hi | *3.45±1.10 Bd* |
| **No Tillage** | Olive Blackwater | 2.03±0.05 st | 4.66±0.05 g | 4.11±0.11 ij | *3.60±1.20 Bc* |
|  | Antep Radish | 2.21±0.03 r-t | 5.90±0.21 d | 3.78±0.05 k | *3.96±1.60 Bb* |
|  | Broccoli | 2.26±0.05 rs | 7.06±0.10 a | 4.10±0.09 ij | *4.48±2.10 Aa* |
|  | Control | 2.46±0.04 qr | 1.74±0.04 u | 5.88±0.50 d | *3.36±1.93 Bd* |
| ***Average of tillage methods*** | *Chisel* | *4.58±1.05 Ab*^***^ | *2.92±0.17 Cc* | *3.26±0.50 Bc* | *3.59±0.98 c* |
|  | *Disc Harrow* | *5.02±1.94 Aa* | *3.53±0.36 Cb* | *4.69±0.40 Ba* | *4.41±1.30 a* |
|  | *No Tillage* | *2.24±0.16 Cc* | *4.84±2.07 Aa* | *4.47±0.89 Bb* | *3.85±1.72 b* |
| ***Average of fertilizers*** | *Olive Blackwater* | *4.16±1.80 Aa* | *3.59±0.85 Cc* | *4.01±1.10 Bb* | *3.92±1.29 b* |
|  | *Antep Radish* | *4.10±2.09 Aa* | *3.95±1.47 Bb* | *3.81±0.52 Cb* | *3.95±1.45 b* |
|  | *Broccoli* | *4.03±1.35 Ca* | *4.53±1.91 Aa* | *4.30±0.45 Ba* | *4.29±1.34 a* |
|  | *Control* | *3.50±1.92 Bb* | *2.98±1.02 Cd* | *4.44±1.22 Aa* | *3.64±1.51 c* |
|  |  |  |  |  |  |
| **Caftaric acid (µg g^-1^ FW)** | | | | | |
| **Tillage** | **Fertilizer** | **Years** | | | ***Average of years*** |
|  |  | **2020** | **2021** | **2022** |  |
| **Chisel** | Olive Blackwater | 4.83±0.17 gh^*^ | 3.15±0.07 j-m | 3.11±0.10 k-n | *3.70±0.86 Cc*^**^ |
|  | Antep Radish | 3.90±0.13 i | 2.91±0.10 l-o | 2.63±0.09 op | *3.15±0.59 Cd* |
|  | Broccoli | 6.39±0.26 e | 3.50±0.08 j | 2.77±0.12 no | *4.22±1.66 Cb* |
|  | Control | 8.00±0.18 c | 3.23±0.11 j-l | 3.41±0.38 jk | *4.88±2.35 Aa* |
| **Disc Harrow** | Olive Blackwater | 7.38±0.26 d | 3.89±0.09 i | 6.23±0.21 e | *5.83±1.55 Aab* |
|  | Antep Radish | 9.38±0.10 b | 3.51±0.11 j | 5.06±0.12 g | *5.99±2.64 Aa* |
|  | Broccoli | 6.38±0.42 e | 4.60±0.06 h | 6.10±0.16 e | *5.70±0.86 Bb* |
|  | Control | 2.29±0.05 pq | 5.57±0.15 f | 4.90±0.09 gh | *4.25±1.50 Bc* |
| **No Tillage** | Olive Blackwater | 2.11±0.07 q | 7.22±0.06 d | 5.08±0.17 g | *4.80±2.22 Bc* |
|  | Antep Radish | 2.55±0.06 op | 9.10±0.30 b | 4.14±0.11 i | *5.26±2.96 Bb* |
|  | Broccoli | 2.35±0.09 pq | 10.75±0.24 a | 5.08±0.13 g | *6.06±3.71 Aa* |
|  | Control | 2.83±0.06 m-o | 2.02±0.06 q | 7.12±0.72 d | *3.99±2.40 Bd* |
| ***Average of tillage methods*** | *Chisel* | *5.78±1.64 Ab*^***^ | *3.20±0.24 Bc* | *2.98±0.36 Cb* | *3.99±1.60 c* |
|  | *Disc Harrow* | *6.36±2.71 Aa* | *4.40±0.82 Cb* | *5.57±0.64 Ba* | *5.44±1.82 a* |
|  | *No Tillage* | *2.46±0.28 Cc* | *7.27±3.43 Aa* | *5.36±1.19 Ba* | *5.03±2.86 b* |
| ***Average of fertilizers*** | *Olive Blackwater* | *4.77±2.29 c* | *4.75±1.88 c* | *4.81±1.37 b* | *4.78±1.81 b* |
|  | *Antep Radish* | *5.28±3.14 Aa* | *5.17±2.96 Ab* | *3.94±1.07 Bc* | *4.80±2.54 b* |
|  | *Broccoli* | *5.04±2.03 Bb* | *6.28±3.38 Aa* | *4.65±1.48 Cb* | *5.32±2.44 a* |
|  | *Control* | *4.37±2.73 Bd* | *3.61±1.57 Cd* | *5.14±1.67 Aa* | *4.37±2.08 c* |
| ^*^Different lowercase letters indicate differences according to the tillage × fertilizer × year interaction (P<0.05). ^**^Different lowercase letters indicate differences among fertilizer means within the same soil tillage method, whereas different uppercase letters indicate differences of the same fertilizer across different soil tillage methods (P<0.05). ^***^Different lowercase letters indicate differences among means in the same column, whereas different uppercase letters indicate differences among means in the same row (P<0.05).  Data are presented as mean + standard deviation (SD). | | | | | |
| **Resveratrol (µg g^-1^ FW)** | | | | | |
| **Tillage** | **Fertilizer** | **Years** | | | ***Average of years*** |
|  |  | **2020** | **2021** | **2022** |  |
| **Chisel** | Olive Blackwater | 3.88±0.04 o-r^*^ | 4.84±0.06 mn | 23.09±0.57 b | *10.60±9.38 Bb*^**^ |
|  | Antep Radish | 3.57±0.14 p-s | 5.07±0.03 m | 15.96±0.25 f | *8.20±5.86 Bd* |
|  | Broccoli | 2.87±0.16 s | 5.06±0.02 m | 18.16±0.75 e | *8.70±7.17 Cc* |
|  | Control | 4.18±0.04 n-q | 6.32±0.12 l | 23.23±0.42 b | *11.24±9.04 Aa* |
| **Disc Harrow** | Olive Blackwater | 4.52±0.03 m-o | 4.82±0.27 mn | 23.29±0.82 b | *10.87±9.32 Ba* |
|  | Antep Radish | 4.50±0.02 m-o | 6.36±0.26 l | 9.50±0.28 i | *6.79±2.20 Cd* |
|  | Broccoli | 4.01±0.10 n-r | 7.21±0.09 k | 17.90±0.55 e | *9.71±6.31 Bb* |
|  | Control | 3.38±0.02 q-s | 7.75±0.28 k | 13.84±0.55 g | *8.32±4.56 Bc* |
| **No Tillage** | Olive Blackwater | 3.31±0.03 rs | 20.97±1.24 d | 11.35±0.31 h | *11.87±7.68 Ac* |
|  | Antep Radish | 4.27±0.04 m-p | 28.35±1.34 a | 10.84±0.04 h | *14.49±10.80 Aa* |
|  | Broccoli | 4.66±0.02 m-o | 21.53±0.67 cd | 13.44±0.35 g | *13.21±7.32 Ab* |
|  | Control | 3.23±0.18 rs | 22.05±0.98 c | 8.73±0.06 j | *11.34±8.39 Ac* |
| ***Average of tillage methods*** | *Chisel* | *3.62±0.51 Cc*^***^ | *5.33±0.61 Bc* | *20.11±3.32 Aa* | *9.69±7.75 b* |
|  | *Disc Harrow* | *4.10±0.48 Ca* | *6.54±1.18 Bb* | *16.13±5.34 Ab* | *8.92±6.10 c* |
|  | *No Tillage* | *3.87±0.64 Cb* | *23.22±3.25 Aa* | *11.09±1.76 Bc* | *12.73±8.37 a* |
| ***Average of fertilizers*** | *Olive Blackwater* | *3.90±0.53 Cb* | *10.21±8.09 Bd* | *19.24±5.94 Aa* | *11.12±8.50 a* |
|  | *Antep Radish* | *4.11±0.42 Ca* | *13.26±11.35 Aa* | *12.10±2.96 Bd* | *9.82±7.72 c* |
|  | *Broccoli* | *3.85±0.79 Cb* | *11.27±7.76 Bc* | *16.50±2.35 Ab* | *10.54±6.96 b* |
|  | *Control* | *3.60±0.45 Cc* | *12.04±7.55 Bb* | *15.26±6.38 Ac* | *10.30±7.43 b* |
| **Pterostilbene (µg g^-1^ FW)** | | | | | |
| **Tillage** | **Fertilizer** | **Years** | | | ***Average of years*** |
|  |  | **2020** | **2021** | **2022** |  |
| **Chisel** | Olive Blackwater | 1.01±0.03 p^*^ | 0.85±0.01 r | 1.30±0.01 l | *1.06±0.20 Ac*^**^ |
|  | Antep Radish | 1.40±0.01 i | 0.77±0.01 t | 0.26±0.01 aa | *0.81±0.50 Cd* |
|  | Broccoli | 1.28±0.01 m | 0.74±0.01 u | 3.38±0.01 a | *1.80±1.21 Aa* |
|  | Control | 0.76±0.00 t | 0.79±0.01 s | 2.40±0.01 c | *1.32±0.81 Bb* |
| **Disc Harrow** | Olive Blackwater | 0.51±0.00 w | 0.46±0.01 x | 1.97±0.02 e | *0.98±0.74 Bd* |
|  | Antep Radish | 0.41±0.01 y | 2.02±0.01 d | 0.73±0.01 u | *1.06±0.74 Bb* |
|  | Broccoli | 0.39±0.00 z | 1.36±0.01 k | 1.90±0.03 f | *1.22±0.66 Ba* |
|  | Control | 0.25±0.01 aa | 1.53±0.01 i | 1.23±0.00 n | *1.00±0.58 Cc* |
| **No Tillage** | Olive Blackwater | 0.21±0.00 bb | 0.86±0.01 r | 1.64±0.01 h | *0.91±0.62 Cd* |
|  | Antep Radish | 0.13±0.00 cc | 0.91±0.01 q | 2.80±0.03 b | *1.28±1.19 Ab* |
|  | Broccoli | 1.07±0.01 o | 0.64±0.01 v | 1.40±0.01 i | *1.03±0.33 Cc* |
|  | Control | 0.85±0.00 r | 3.39±0.03 a | 1.78±0.02 g | *2.01±1.11 Aa* |
| ***Average of tillage methods*** | *Chisel* | *1.11±0.26 Ba*^***^ | *0.79±0.04 Cc* | *1.84±1.22 Ab* | *1.25±0.83 b* |
|  | *Disc Harrow* | *0.39±0.10 Cc* | *1.34±0.59 Bb* | *1.46±0.53 Ac* | *1.06±0.66 c* |
|  | *No Tillage* | *0.56±0.42 Cb* | *1.45±1.18 Ba* | *1.90±0.56 Aa* | *1.31±0.95 a* |
| ***Average of fertilizers*** | *Olive Blackwater* | *0.58±0.35 Cd* | *0.73±0.20 Bd* | *1.64±0.29 Ac* | *0.98±0.55 d* |
|  | *Antep Radish* | *0.65±0.58 Cb* | *1.23±0.59 Bb* | *1.26±1.17 Ad* | *1.05±0.85 c* |
|  | *Broccoli* | *0.91±0.40 Ba* | *0.91±0.34 Bc* | *2.22±0.89 Aa* | *1.35±0.85 b* |
|  | *Control* | *0.62±0.28 Cc* | *1.90±1.16 Aa* | *1.80±0.51 Bb* | *1.44±0.93 a* |
| ^*^Different lowercase letters indicate differences according to the tillage × fertilizer × year interaction (P<0.05). ^**^Different lowercase letters indicate differences among fertilizer means within the same soil tillage method, whereas different uppercase letters indicate differences of the same fertilizer across different soil tillage methods (P<0.05). ^***^Different lowercase letters indicate differences among means in the same column, whereas different uppercase letters indicate differences among means in the same row (P<0.05).  Data are presented as mean + standard deviation (SD). | | | | | |
|  |  |  |  |  |  |
| **Piceid (µg g^-1^ FW)** | | | | | |
| **Tillage** | **Fertilizer** | **Years** | | | ***Average of years*** |
|  |  | **2020** | **2021** | **2022** |  |
| **Chisel** | Olive Blackwater | 4.84±0.09 d^*^ | 0.46±0.04 p-r | 7.57±0.16 a | *4.29±3.11 Aa*^**^ |
|  | Antep Radish | 3.83±0.36 e | 0.12±0.01 vw | 5.86±0.08 b | *3.27±2.53 Ab* |
|  | Broccoli | 0.43±0.01 p-s | 0.52±0.08 p | 5.47±0.18 c | *2.14±2.50 Ac* |
|  | Control | 0.34±0.01 q-u | 1.03±0.06 n | 3.63±0.04 f | *1.67±1.50 Ad* |
| **Disc Harrow** | Olive Blackwater | 0.29±0.02 r-v | 0.40±0.01 p-t | 2.80±0.20 i | *1.17±1.23 Ca* |
|  | Antep Radish | 0.18±0.00 u-w | 0.33±0.01 q-u | 3.19±0.02 h | *1.23±1.47 Ca* |
|  | Broccoli | 0.14±0.00 vw | 0.28±0.01 s-w | 1.59±0.01 l | *0.67±0.69 Cc* |
|  | Control | 0.11±0.01 w | 1.35±0.26 m | 1.22±0.01 m | *0.89±0.61 Bb* |
| **No Tillage** | Olive Blackwater | 0.35±0.06 p-u | 3.44±0.02 g | 0.25±0.01 t-w | *1.35±1.57 Bd* |
|  | Antep Radish | 0.84±0.04 o | 3.19±0.01 h | 3.17±0.02 h | *2.40±1.17 Ba* |
|  | Broccoli | 0.50±0.00 pq | 3.14±0.03 h | 2.26±0.01 j | *1.97±1.16 Bb* |
|  | Control | 0.50±0.01 pq | 2.86±0.09 i | 1.86±0.02 k | *1.74±1.03 Ac* |
| ***Average of tillage methods*** | *Chisel* | *2.36±2.10 Ba*^***^ | *0.53±0.34 Cb* | *5.63±1.47 Aa* | *2.84±2.58 a* |
|  | *Disc Harrow* | *0.18±0.07 Cc* | *0.59±0.47 Bb* | *2.20±0.86 Ab* | *0.99±1.04 c* |
|  | *No Tillage* | *0.55±0.19 Cb* | *3.16±0.22 Aa* | *1.88±1.10 Bc* | *1.86±1.26 b* |
| ***Average of fertilizers*** | *Olive Blackwater* | *1.83±2.26 Ba* | *1.44±1.51 Cb* | *3.54±3.22 Ab* | *2.27±2.52 a* |
|  | *Antep Radish* | *1.62±1.69 Bb* | *1.21±1.49 Cd* | *4.07±1.34 Aa* | *2.30±1.94 a* |
|  | *Broccoli* | *0.36±0.17 Cc* | *1.31±1.37 Bc* | *3.11±1.80 Ac* | *1.59±1.71 b* |
|  | *Control* | *0.32±0.17 Cc* | *1.75±0.86 Ba* | *2.24±1.08 Ad* | *1.43±1.13 c* |
|  |  |  |  |  |  |
|  |  |  |  |  |  |
| **Viniferin (µg g^-1^ FW)** | | | | | |
| **Tillage** | **Fertilizer** | **Years** | | | ***Average of years*** |
|  |  | **2020** | **2021** | **2022** |  |
| **Chisel** | Olive Blackwater | 5.42±0.04 r^*^ | 11.77±0.38 k | 21.32±0.09 f | *12.84±6.94 Cc*^**^ |
|  | Antep Radish | 5.29±0.14 r | 11.21±0.26 lm | 20.11±0.08 g | *12.20±6.46 Cd* |
|  | Broccoli | 5.52±0.03 r | 11.79±0.46 k | 24.55±0.08 cd | *13.96±8.40 Cb* |
|  | Control | 8.79±0.46 o | 10.84±0.28 m | 23.99±0.19 e | *14.54±7.15 Ba* |
| **Disc Harrow** | Olive Blackwater | 9.81±0.18 n | 10.86±0.27 m | 20.23±0.32 g | *13.64±4.97 Bc* |
|  | Antep Radish | 11.17±0.42 lm | 6.42±0.09 q | 21.06±0.57 f | *12.88±6.48 Bd* |
|  | Broccoli | 9.17±0.13 o | 11.65±0.69 kl | 25.01±0.41 bc | *15.28±7.39 Ab* |
|  | Control | 9.91±0.27 n | 15.42±0.35 i | 25.33±0.42 b | *16.89±6.78 Aa* |
| **No Tillage** | Olive Blackwater | 8.00±0.40 p | 16.22±0.63 h | 26.94±0.08 a | *17.05±8.23 Aa* |
|  | Antep Radish | 5.44±0.05 r | 14.91±0.39 i | 24.04±0.39 de | *14.80±8.06 Ab* |
|  | Broccoli | 6.33±0.05 q | 13.22±0.51 j | 23.65±0.10 e | *14.40±7.56 Bc* |
|  | Control | 5.65±0.13 r | 12.20±0.26 k | 16.24±0.05 h | *11.36±4.63 Cd* |
| ***Average of tillage methods*** | *Chisel* | *6.26±1.54 Cb*^***^ | *11.41±0.51 Bb* | *22.49±1.92 Ab* | *13.38±7.01 c* |
|  | *Disc Harrow* | *10.01±0.79 Ca* | *11.09±3.36 Bb* | *22.91±2.41 Aa* | *14.67±6.38 a* |
|  | *No Tillage* | *6.35±1.07 Cb* | *14.14±1.66 Ba* | *22.72±4.13 Aab* | *14.40±7.25 b* |
| ***Average of fertilizers*** | *Olive Blackwater* | *7.74±1.93 Cb* | *12.95±2.51 Ba* | *22.83±3.12 Ab* | *14.51±6.84 a* |
|  | *Antep Radish* | *7.30±2.91 Cc* | *10.85±3.69 Bc* | *21.74±1.81 Ac* | *13.30±6.86 c* |
|  | *Broccoli* | *7.01±1.66 Cd* | *12.22±0.89 Bb* | *24.40±0.64 Aa* | *14.54±7.51 a* |
|  | *Control* | *8.12±1.93 Ca* | *12.82±2.05 Ba* | *21.85±4.26 Ac* | *14.26±6.46 b* |
| ^*^Different lowercase letters indicate differences according to the tillage × fertilizer × year interaction (P<0.05). ^**^Different lowercase letters indicate differences among fertilizer means within the same soil tillage method, whereas different uppercase letters indicate differences of the same fertilizer across different soil tillage methods (P<0.05). ^***^Different lowercase letters indicate differences among means in the same column, whereas different uppercase letters indicate differences among means in the same row (P<0.05).  Data are presented as mean + standard deviation (SD). | | | | | |
| **Tyrosol (µg g^-1^ FW)** | | | | | |
| **Tillage** | **Fertilizer** | **Years** | | | ***Average of years*** |
|  |  | **2020** | **2021** | **2022** |  |
| **Chisel** | Olive Blackwater | 6.02±0.23 g^*^ | 4.51±0.13 jk | 4.48±0.17 kl | *5.00±0.78 Cc*^**^ |
|  | Antep Radish | 4.86±0.15 ij | 4.12±0.13 lm | 3.73±0.11 no | *4.24±0.51 Cd* |
|  | Broccoli | 8.02±0.29 e | 5.02±0.14 i | 4.00±0.19 m-o | *5.68±1.82 Cb* |
|  | Control | 10.07±0.29 c | 4.58±0.14 jk | 4.44±0.44 kl | *6.36±2.79 Aa* |
| **Disc Harrow** | Olive Blackwater | 9.19±0.29 d | 5.58±0.16 h | 7.78±0.36 e | *7.52±1.59 Aa* |
|  | Antep Radish | 11.36±0.29 b | 4.44±0.03 kl | 5.77±0.14 gh | *7.19±3.18 Ab* |
|  | Broccoli | 7.34±0.42 f | 4.98±0.03 i | 7.17±0.25 f | *6.50±1.16 Bc* |
|  | Control | 3.28±0.10 q | 6.10±0.14 g | 5.58±0.10 h | *4.99±1.30 Bd* |
| **No Tillage** | Olive Blackwater | 3.00±0.09 qr | 8.01±0.03 e | 6.09±0.23 g | *5.70±2.20 Bc* |
|  | Antep Radish | 3.65±0.11 op | 10.05±0.27 c | 4.94±0.10 i | *6.21±2.94 Bb* |
|  | Broccoli | 3.33±0.10 pq | 11.87±0.34 a | 6.05±0.20 g | *7.08±3.78 Aa* |
|  | Control | 4.06±0.11 mn | 2.90±0.11 r | 4.45±0.02 kl | *3.80±0.70 Cd* |
| ***Average of tillage methods*** | *Chisel* | *7.24±2.08 Ab*^***^ | *4.56±0.35 Bc* | *4.16±0.39 Cc* | *5.32±1.84 c* |
|  | *Disc Harrow* | *7.79±3.11 Aa* | *5.28±0.66 Cb* | *6.57±0.99 Ba* | *6.55±2.14 a* |
|  | *No Tillage* | *3.51±0.42 Cc* | *8.21±3.51 Aa* | *5.38±0.75 Bb* | *5.70±2.82 b* |
| ***Average of fertilizers*** | *Olive Blackwater* | *6.07±2.69 c* | *6.04±1.56 c* | *6.11±1.45 a* | *6.07±1.90 b* |
|  | *Antep Radish* | *6.62±3.60 Aa* | *6.21±2.89 Bb* | *4.82±0.89 Cc* | *5.88±2.72 c* |
|  | *Broccoli* | *6.23±2.21 Bb* | *7.29±3.44 Aa* | *5.74±1.40 Cb* | *6.42±2.49 a* |
|  | *Control* | *5.80±3.22 Ad* | *4.53±1.39 Cd* | *4.82±0.61 Bc* | *5.05±2.05 d* |
|  |  |  |  |  |  |
|  |  |  |  |  |  |
| **Catechin (µg g^-1^ FW)** | | | | | |
| **Tillage** | **Fertilizer** | **Years** | | | ***Average of years*** |
|  |  | **2020** | **2021** | **2022** |  |
| **Chisel** | Olive Blackwater | 4.93±0.16 i-k^*^ | 3.74±0.09 n-q | 3.71±0.13 o-q | *4.13±0.62 Cc*^**^ |
|  | Antep Radish | 4.22±0.19 l-n | 3.58±0.16 pq | 5.03±0.53 ij | *4.27±0.70 Cc* |
|  | Broccoli | 7.01±0.34 de | 4.16±0.10 l-o | 6.75±0.52 e | *5.97±1.40 a* |
|  | Control | 8.34±0.21 bc | 3.98±0.18 m-p | 4.31±0.51 lm | *5.54±2.12 Ab* |
| **Disc Harrow** | Olive Blackwater | 7.97±0.36 c | 4.62±0.11 j-l | 7.21±0.27 de | *6.60±1.54 Aa* |
|  | Antep Radish | 9.42±0.20 a | 3.69±0.05 o-q | 6.16±0.21 f | *6.42±2.50 Aa* |
|  | Broccoli | 6.07±0.30 fg | 4.13±0.04 l-o | 7.36±0.25 d | *5.86±1.42 b* |
|  | Control | 2.72±0.07 st | 5.27±0.19 hi | 5.36±0.15 hi | *4.45±1.30 Bc* |
| **No Tillage** | Olive Blackwater | 2.60±0.12 st | 6.89±0.11 de | 5.63±0.21 gh | *5.04±1.91 Bc* |
|  | Antep Radish | 3.02±0.08 rs | 8.75±0.35 b | 4.51±0.16 kl | *5.43±2.58 Bb* |
|  | Broccoli | 2.89±0.13 rs | 9.84±0.24 a | 5.61±0.19 gh | *6.11±3.04 a* |
|  | Control | 3.36±0.09 qr | 2.39±0.08 t | 7.99±0.85 c | *4.58±2.63 Bd* |
| ***Average of tillage methods*** | *Chisel* | *6.13±1.73 Ab*^***^ | *3.86±0.26 Cc* | *4.95±1.25 Bc* | *4.98±1.53 c* |
|  | *Disc Harrow* | *6.55±2.63 Aa* | *4.43±0.62 Bb* | *6.52±0.87 Aa* | *5.83±1.88 a* |
|  | *No Tillage* | *2.97±0.30 Cc* | *6.97±2.98 Aa* | *5.93±1.38 Bb* | *5.29±2.52 b* |
| ***Average of fertilizers*** | *Olive Blackwater* | *5.17±2.34 Bb* | *5.08±1.41 Bc* | *5.51±1.53 Abc* | *5.26±1.75 b* |
|  | *Antep Radish* | *5.55±2.95 Aa* | *5.34±2.57 ABb* | *5.23±0.79 Bc* | *5.38±2.22 b* |
|  | *Broccoli* | *5.33±1.88 Cb* | *6.04±2.85 Ba* | *6.57±0.83 Aa* | *5.98±2.02 a* |
|  | *Control* | *4.81±2.67 Bc* | *3.88±1.26 Cd* | *5.89±1.72 Ab* | *4.86±2.07 c* |
| ^*^Different lowercase letters indicate differences according to the tillage × fertilizer × year interaction (P<0.05). ^**^Different lowercase letters indicate differences among fertilizer means within the same soil tillage method, whereas different uppercase letters indicate differences of the same fertilizer across different soil tillage methods (P<0.05). ^***^Different lowercase letters indicate differences among means in the same column, whereas different uppercase letters indicate differences among means in the same row (P<0.05).  Data are presented as mean + standard deviation (SD). | | | | | |
| **Epicatechin (µg g^-1^ FW)** | | | | | |
| **Tillage** | **Fertilizer** | **Years** | | | ***Average of years*** |
|  |  | **2020** | **2021** | **2022** |  |
| **Chisel** | Olive Blackwater | 3.33±0.14 h^*^ | 1.81±0.03 pq | 1.78±0.05 pq | *2.31±0.77 Cb*^**^ |
|  | Antep Radish | 2.31±0.04 lm | 1.75±0.03 pq | 2.61±0.25 jk | *2.22±0.40 Cb* |
|  | Broccoli | 3.61±0.14 g | 2.05±0.04 no | 3.60±0.28 g | *3.09±0.79 Ba* |
|  | Control | 5.05±0.09 cd | 1.98±0.03 n-p | 1.85±0.17 op | *2.96±1.57 Aa* |
| **Disc Harrow** | Olive Blackwater | 4.87±0.08 d | 2.32±0.04 lm | 3.37±0.12 h | *3.52±1.11 Ab* |
|  | Antep Radish | 5.99±0.04 b | 2.28±0.04 m | 2.77±0.03 ij | *3.68±1.75 Aa* |
|  | Broccoli | 4.38±0.34 e | 2.91±0.02 i | 3.31±0.08 h | *3.53±0.68 Ab* |
|  | Control | 1.25±0.02 st | 3.32±0.03 h | 2.63±0.02 jk | *2.40±0.91 Bc* |
| **No Tillage** | Olive Blackwater | 1.20±0.02 st | 3.92±0.02 f | 2.53±0.07 kl | *2.55±1.18 Bc* |
|  | Antep Radish | 1.41±0.03 rs | 5.12±0.16 c | 2.20±0.02 mn | *2.91±1.69 Bb* |
|  | Broccoli | 1.36±0.02 s | 6.69±0.11 a | 2.57±0.06 jk | *3.54±2.42 Aa* |
|  | Control | 1.60±0.03 qr | 1.10±0.03 t | 4.04±0.41 f | *2.25±1.38 Cd* |
| ***Average of tillage methods*** | *Chisel* | *3.58±1.03 Ab*^***^ | *1.90±0.13 Cc* | *2.46±0.79 Bc* | *2.64±1.02 c* |
|  | *Disc Harrow* | *4.12±1.85 Aa* | *2.71±0.45 Cb* | *3.02±0.34 Ba* | *3.28±1.25 a* |
|  | *No Tillage* | *1.40±0.15 Cc* | *4.21±2.14 Aa* | *2.84±0.76 Bb* | *2.81±1.73 b* |
| ***Average of fertilizers*** | *Olive Blackwater* | *3.14±1.60 Aa* | *2.69±0.95 Bc* | *2.56±0.69 Cc* | *2.79±1.13 c* |
|  | *Antep Radish* | *3.24±2.10 Aa* | *3.05±1.57 Bb* | *2.53±0.29 Cc* | *2.94±1.50 b* |
|  | *Broccoli* | *3.12±1.37 Ba* | *3.88±2.14 Aa* | *3.16±0.48 Ba* | *3.39±1.48 a* |
|  | *Control* | *2.63±1.82 Bb* | *2.13±0.97 Cd* | *2.84±0.99 Ab* | *2.54±1.30 d* |
|  |  |  |  |  |  |
|  |  |  |  |  |  |
| **Rutin (µg g^-1^ FW)** | | | | | |
| **Tillage** | **Fertilizer** | **Years** | | | ***Average of years*** |
|  |  | **2020** | **2021** | **2022** |  |
| **Chisel** | Olive Blackwater | 2.21±0.06 k^*^ | 1.53±0.02 o | 1.51±0.04 op | *1.75±0.35 Cc*^**^ |
|  | Antep Radish | 1.71±0.06 n | 1.35±0.04 qr | 1.22±0.03 r-t | *1.43±0.23 Cd* |
|  | Broccoli | 2.79±0.11 hi | 1.70±0.03 n | 1.34±0.05 qr | *1.94±0.66 Cb* |
|  | Control | 3.67±0.06 d | 1.50±0.05 op | 2.08±0.28 l | *2.42±0.99 Aa* |
| **Disc Harrow** | Olive Blackwater | 3.23±0.10 f | 1.89±0.03 m | 4.48±0.12 b | *3.20±1.13 Aa* |
|  | Antep Radish | 4.25±0.04 c | 1.66±0.05 n | 3.49±0.07 e | *3.13±1.16 Aa* |
|  | Broccoli | 2.71±0.17 ij | 2.03±0.04 l | 4.41±0.08 b | *3.05±1.07 Bb* |
|  | Control | 1.11±0.02 s-u | 2.31±0.06 k | 3.38±0.05 e | *2.27±0.98 Bc* |
| **No Tillage** | Olive Blackwater | 0.98±0.03 u | 3.00±0.02 g | 3.66±0.10 d | *2.54±1.21 Bc* |
|  | Antep Radish | 1.24±0.02 q-s | 3.77±0.11 d | 2.85±0.06 h | *2.62±1.11 Bb* |
|  | Broccoli | 1.09±0.03 tu | 4.67±0.07 a | 3.67±0.06 d | *3.14±1.60 Aa* |
|  | Control | 1.37±0.03 pq | 0.98±0.02 u | 2.64±0.01 j | *1.66±0.75 Cd* |
| ***Average of tillage methods*** | *Chisel* | *2.60±0.76 Ab*^***^ | *1.52±0.13 Bc* | *1.54±0.37 Bc* | *1.88±0.70 c* |
|  | *Disc Harrow* | *2.83±1.19 Ba* | *1.97±0.25 Cb* | *3.94±0.53 Aa* | *2.91±1.10 a* |
|  | *No Tillage* | *1.17±0.16 Cc* | *3.10±1.42 Ba* | *3.21±0.49 Ab* | *2.49±1.27 b* |
| ***Average of fertilizers*** | *Olive Blackwater* | *2.14±0.98 Bb* | *2.14±0.66 Bc* | *3.22±1.33 Aa* | *2.50±1.12 b* |
|  | *Antep Radish* | *2.40±1.40 Ba* | *2.26±1.14 Cb* | *2.52±1.02 Ac* | *2.39±1.16 c* |
|  | *Broccoli* | *2.20±0.84 Cb* | *2.80±1.41 Ba* | *3.14±1.39 Aa* | *2.71±1.26 a* |
|  | *Control* | *2.05±1.22 Bc* | *1.60±0.58 Cd* | *2.70±0.58 Ab* | *2.12±0.94 d* |
| ^*^Different lowercase letters indicate differences according to the tillage × fertilizer × year interaction (P<0.05). ^**^Different lowercase letters indicate differences among fertilizer means within the same soil tillage method, whereas different uppercase letters indicate differences of the same fertilizer across different soil tillage methods (P<0.05). ^***^Different lowercase letters indicate differences among means in the same column, whereas different uppercase letters indicate differences among means in the same row (P<0.05).  Data are presented as mean + standard deviation (SD). | | | | | |
| **Quercetin (µg g^-1^ FW)** | | | | | |
| **Tillage** | **Fertilizer** | **Years** | | | ***Average of years*** |
|  |  | **2020** | **2021** | **2022** |  |
| **Chisel** | Olive Blackwater | 1.89±0.03 st^*^ | 2.34±0.03 j-m | 2.38±0.04 i-l | *2.21±0.24 Cc*^**^ |
|  | Antep Radish | 2.10±0.04 o-r | 2.04±0.03 p-t | 2.72±0.17 h | *2.29±0.34 Cc* |
|  | Broccoli | 2.39±0.02 i-l | 2.29±0.02 k-n | 3.26±0.16 g | *2.65±0.47 Ca* |
|  | Control | 2.14±0.02 n-q | 2.00±0.03 q-t | 3.36±0.37 g | *2.50±0.67 Cb* |
| **Disc Harrow** | Olive Blackwater | 1.86±0.03 t | 2.25±0.03 l-o | 6.26±0.19 a | *3.46±2.11 Ba* |
|  | Antep Radish | 2.12±0.02 n-r | 1.95±0.03 r-t | 4.53±0.04 e | *2.87±1.25 Bc* |
|  | Broccoli | 2.03±0.06 p-t | 2.06±0.01 p-s | 5.14±0.11 c | *3.08±1.55 Bb* |
|  | Control | 2.49±0.03 ij | 2.14±0.04 n-q | 4.74±0.20 d | *3.12±1.23 Ab* |
| **No Tillage** | Olive Blackwater | 2.17±0.03 m-q | 2.52±0.01 i | 6.35±0.15 a | *3.68±2.01 Aa* |
|  | Antep Radish | 2.44±0.03 i-k | 2.43±0.02 i-l | 5.00±0.03 c | *3.29±1.28 Ab* |
|  | Broccoli | 2.12±0.04 n-r | 2.20±0.03 m-p | 5.59±0.10 b | *3.31±1.72 Ab* |
|  | Control | 2.39±0.03 i-l | 2.18±0.04 m-q | 4.09±0.04 f | *2.89±0.91 Bc* |
| ***Average of tillage methods*** | *Chisel* | *2.13±0.18 Bb*^***^ | *2.17±0.16 Bb* | *2.93±0.45 Ab* | *2.41±0.47 c* |
|  | *Disc Harrow* | *2.13±0.24 Bb* | *2.10±0.12 Bc* | *5.17±0.71 Aa* | *3.13±1.52 b* |
|  | *No Tillage* | *2.28±0.14 Ca* | *2.33±0.16 Ba* | *5.26±0.87 Aa* | *3.29±1.50 a* |
| ***Average of fertilizers*** | *Olive Blackwater* | *1.98±0.15 Cd* | *2.37±0.12 Ba* | *5.00±1.97 Aa* | *3.12±1.75 a* |
|  | *Antep Radish* | *2.22±0.16 Bb* | *2.14±0.22 Cc* | *4.08±1.04 Ac* | *2.81±1.09 c* |
|  | *Broccoli* | *2.18±0.16 Bc* | *2.19±0.10 Bb* | *4.66±1.08 Ab* | *3.01±1.34 b* |
|  | *Control* | *2.34±0.16 Ba* | *2.11±0.09 Cd* | *4.06±0.64 Ac* | *2.84±0.96 c* |
|  |  |  |  |  |  |
|  |  |  |  |  |  |
| **Myricetin (µg g^-1^ FW)** | | | | | |
| **Tillage** | **Fertilizer** | **Years** | | | ***Average of years*** |
|  |  | **2020** | **2021** | **2022** |  |
| **Chisel** | Olive Blackwater | 1.68±0.06 jk^*^ | 1.34±0.05 m-p | 1.35±0.07 m-p | *1.46±0.17 Cc*^**^ |
|  | Antep Radish | 1.37±0.05 m-o | 1.02±0.03 q-u | 0.92±0.03 s-v | *1.10±0.21 Cd* |
|  | Broccoli | 2.62±0.06 f | 1.50±0.06 k-m | 1.20±0.07 o-q | *1.77±0.65 Cb* |
|  | Control | 3.42±0.13 d | 1.13±0.05 p-s | 1.05±0.10 q-t | *1.87±1.17 Aa* |
| **Disc Harrow** | Olive Blackwater | 2.59±0.10 f | 1.66±0.07 jk | 2.18±0.10 g | *2.15±0.41 Aa* |
|  | Antep Radish | 3.98±0.11 b | 1.23±0.04 n-q | 1.44±0.03 l-n | *2.21±1.33 Aa* |
|  | Broccoli | 2.22±0.14 g | 1.60±0.02 j-l | 2.14±0.10 gh | *1.99±0.30 Bb* |
|  | Control | 0.98±0.04 r-u | 1.95±0.06 hi | 1.39±0.03 m-o | *1.44±0.42 Bc* |
| **No Tillage** | Olive Blackwater | 0.74±0.03 v | 3.03±0.04 e | 1.78±0.09 ij | *1.85±0.99 Bb* |
|  | Antep Radish | 1.09±0.04 q-s | 3.76±0.06 c | 1.18±0.03 o-r | *2.01±1.31 Bb* |
|  | Broccoli | 0.82±0.03 uv | 4.59±0.18 a | 1.78±0.08 ij | *2.40±1.70 Aa* |
|  | Control | 1.21±0.05 o-q | 0.87±0.04 t-v | 3.59±0.55 cd | *1.89±1.32 Ab* |
| ***Average of tillage methods*** | *Chisel* | *2.27±0.85 Ab*^***^ | *1.25±0.20 Bc* | *1.13±0.18 Cc* | *1.55±0.72 c* |
|  | *Disc Harrow* | *2.44±1.12 Aa* | *1.61±0.27 Cb* | *1.79±0.39 Bb* | *1.95±0.77 b* |
|  | *No Tillage* | *0.97±0.20 Cc* | *3.06±1.45 Aa* | *2.08±0.98 Ba* | *2.04±1.31 a* |
| ***Average of fertilizers*** | *Olive Blackwater* | *1.67±0.80 Cc* | *2.01±0.78 Ab* | *1.77±0.37 Bb* | *1.82±0.67 b* |
|  | *Antep Radish* | *2.15±1.38 Aa* | *2.00±1.32 Bb* | *1.18±0.23 Cc* | *1.78±1.15 bc* |
|  | *Broccoli* | *1.89±0.82 Bb* | *2.56±1.53 Aa* | *1.71±0.41 Cb* | *2.05±1.06 a* |
|  | *Control* | *1.87±1.17 Ab* | *1.32±0.49 Bc* | *2.01±1.23 Aa* | *1.73±1.03 c* |
| ^*^Different lowercase letters indicate differences according to the tillage × fertilizer × year interaction (P<0.05). ^**^Different lowercase letters indicate differences among fertilizer means within the same soil tillage method, whereas different uppercase letters indicate differences of the same fertilizer across different soil tillage methods (P<0.05). ^***^Different lowercase letters indicate differences among means in the same column, whereas different uppercase letters indicate differences among means in the same row (P<0.05).  Data are presented as mean + standard deviation (SD) | | | | | |
| **Delphinidin-3-*O*-glucoside (%)** | | | | | |
| **Tillage** | **Fertilizer** | **Years** | | | ***Average of years*** |
|  |  | **2020** | **2021** | **2022** |  |
| **Chisel** | Olive Blackwater | 4.04±0.03 f^*^ | 2.26±0.02 q | 2.28±0.02 q | *2.86±0.88 Bc*^**^ |
|  | Antep Radish | 2.13±0.02 r | 1.58±0.01 y | 1.08±0.01 cc | *1.60±0.46 Cd* |
|  | Broccoli | 3.39±0.02 j | 2.52±0.01 n | 3.53±0.05 i | *3.15±0.48 Bb* |
|  | Control | 6.65±0.03 b | 1.76±0.01 v | 1.42±0.02 z | *3.28±2.54 Aa* |
| **Disc Harrow** | Olive Blackwater | 4.65±0.04 d | 2.80±0.01 m | 4.62±0.03 d | *4.02±0.92 Ac* |
|  | Antep Radish | 7.40±0.04 a | 1.95±0.02 t | 2.87±0.04 l | *4.07±2.53 Ab* |
|  | Broccoli | 6.05±0.05 c | 1.99±0.02 t | 4.30±0.05 e | *4.11±1.77 Aa* |
|  | Control | 1.65±0.01 x | 1.80±0.01 uv | 2.93±0.02 k | *2.12±0.61 Bd* |
| **No Tillage** | Olive Blackwater | 1.15±0.01 bb | 1.09±0.01 cc | 3.78±0.03 g | *2.01±1.33 Cc* |
|  | Antep Radish | 1.83±0.01 u | 1.40±0.01 z | 2.33±0.04 p | *1.85±0.40 Bd* |
|  | Broccoli | 1.28±0.01 aa | 2.16±0.02 r | 3.57±0.05 h | *2.34±1.00 Ca* |
|  | Control | 2.04±0.01 s | 1.70±0.05 w | 2.45±0.02 o | *2.06±0.33 Cb* |
| ***Average of tillage methods*** | *Chisel* | *4.05±1.72 Ab*^***^ | *2.03±0.39 Cb* | *2.08±0.99 Bc* | *2.72±1.48 b* |
|  | *Disc Harrow* | *4.94±2.23 Aa* | *2.13±0.41 Ca* | *3.68±0.82 Ba* | *3.58±1.78 a* |
|  | *No Tillage* | *1.57±0.39 Bc* | *1.59±0.41 Bc* | *3.03±0.68 Ab* | *2.07±0.85 c* |
| ***Average of fertilizers*** | *Olive Blackwater* | *3.28±1.62 Bd* | *2.05±0.76 Cb* | *3.56±1.03 Ab* | *2.96±1.32 b* |
|  | *Antep Radish* | *3.79±2.71 Aa* | *1.65±0.24 Cd* | *2.09±0.80 Bd* | *2.51±1.83 c* |
|  | *Broccoli* | *3.58±2.07 Bb* | *2.22±0.23 Ca* | *3.80±0.38 Aa* | *3.20±1.37 a* |
|  | *Control* | *3.45±2.41 Ac* | *1.75±0.05 Cc* | *2.27±0.67 Bc* | *2.49±1.56 d* |
|  |  |  |  |  |  |
|  |  |  |  |  |  |
| **Cyanidin-3-*O*-glucoside (%)** | | | | | |
| **Tillage** | **Fertilizer** | **Years** | | | ***Average of years*** |
|  |  | **2020** | **2021** | **2022** |  |
| **Chisel** | Olive Blackwater | 2.34±0.02 j^*^ | 1.42±0.02 op | 1.44±0.03 o | *1.73±0.45 Bb*^**^ |
|  | Antep Radish | 1.38±0.01 p | 1.18±0.01 r | 0.81±0.01 w | *1.12±0.25 Cd* |
|  | Broccoli | 1.85±0.03 k | 1.58±0.02 n | 1.69±0.05 m | *1.71±0.12 Bc* |
|  | Control | 3.14±0.04 e | 1.31±0.01 q | 1.06±0.00 s | *1.84±0.99 Aa* |
| **Disc Harrow** | Olive Blackwater | 2.60±0.02 h | 1.76±0.03 l | 4.00±0.07 a | *2.79±0.98 Aa* |
|  | Antep Radish | 3.50±0.05 c | 1.46±0.01 o | 2.93±0.01 g | *2.63±0.91 Ab* |
|  | Broccoli | 3.04±0.02 f | 1.15±0.01 r | 3.74±0.09 b | *2.64±1.16 Ab* |
|  | Control | 1.03±0.02 st | 0.93±0.01 v | 3.00±0.02 f | *1.65±1.01 Bc* |
| **No Tillage** | Olive Blackwater | 0.86±0.01 w | 0.71±0.01 x | 3.27±0.05 d | *1.61±1.25 Cb* |
|  | Antep Radish | 1.15±0.02 r | 0.84±0.01 w | 2.37±0.01 j | *1.45±0.70 Bc* |
|  | Broccoli | 0.95±0.01 uv | 1.18±0.02 r | 3.10±0.07 e | *1.75±1.02 Ba* |
|  | Control | 1.28±0.02 q | 1.00±0.00 tu | 2.52±0.02 i | *1.60±0.70 Cb* |
| ***Average of tillage methods*** | *Chisel* | *2.18±0.68 Ab*^***^ | *1.37±0.16 Ba* | *1.25±0.36 Cc* | *1.60±0.61 b* |
|  | *Disc Harrow* | *2.54±0.97 Ba* | *1.32±0.33 Cb* | *3.42±0.48 Aa* | *2.43±1.08 a* |
|  | *No Tillage* | *1.06±0.17 Bc* | *0.93±0.18 Cc* | *2.82±0.40 Ab* | *1.60±0.91 b* |
| ***Average of fertilizers*** | *Olive Blackwater* | *1.93±0.82 Bb* | *1.30±0.46 Ca* | *2.91±1.14 Aa* | *2.05±1.06 a* |
|  | *Antep Radish* | *2.01±1.12 Ba* | *1.16±0.27 Cb* | *2.04±0.95 Ad* | *1.73±0.93 b* |
|  | *Broccoli* | *1.95±0.91 Bb* | *1.30±0.21 Ca* | *2.84±0.91 Ab* | *2.03±0.97 a* |
|  | *Control* | *1.82±1.00 Bc* | *1.08±0.18 Cc* | *2.19±0.88 Ac* | *1.70±0.88 c* |
| ^*^Different lowercase letters indicate differences according to the tillage × fertilizer × year interaction (P<0.05). ^**^Different lowercase letters indicate differences among fertilizer means within the same soil tillage method, whereas different uppercase letters indicate differences of the same fertilizer across different soil tillage methods (P<0.05). ^***^Different lowercase letters indicate differences among means in the same column, whereas different uppercase letters indicate differences among means in the same row (P<0.05).  Data are presented as mean + standard deviation (SD). | | | | | |
| **Petunidin-3-*O*-glucoside (%)** | | | | | |
| **Tillage** | **Fertilizer** | **Years** | | | ***Average of years*** |
|  |  | **2020** | **2021** | **2022** |  |
| **Chisel** | Olive Blackwater | 8.42±0.03 f^*^ | 4.71±0.05 q | 4.77±0.08 q | *5.97±1.84 Bb*^**^ |
|  | Antep Radish | 5.00±0.02 p | 3.97±0.02 t | 2.66±0.01 aa | *3.88±1.01 Bd* |
|  | Broccoli | 6.66±0.07 i | 5.29±0.06 o | 4.51±0.05 r | *5.49±0.94 Bc* |
|  | Control | 11.02±0.11 c | 4.45±0.02 r | 3.55±0.03 v | *6.34±3.53 Aa* |
| **Disc Harrow** | Olive Blackwater | 9.28±0.03 e | 5.93±0.06 l | 9.56±0.13 d | *8.26±1.75 Aa* |
|  | Antep Radish | 12.37±0.13 a | 5.00±0.02 p | 6.31±0.07 k | *7.89±3.40 Ac* |
|  | Broccoli | 11.90±0.04 b | 3.77±0.01 u | 8.37±0.17 f | *8.02±3.53 Ab* |
|  | Control | 3.34±0.04 w | 3.02±0.04 y | 6.47±0.02 j | *4.27±1.65 Bd* |
| **No Tillage** | Olive Blackwater | 2.81±0.01 z | 2.19±0.02 cc | 7.15±0.09 g | *4.05±2.34 Cc* |
|  | Antep Radish | 3.74±0.04 u | 2.42±0.03 bb | 5.39±0.06 n | *3.85±1.29 Bd* |
|  | Broccoli | 3.15±0.01 x | 3.71±0.04 u | 6.88±0.14 h | *4.58±1.74 Ca* |
|  | Control | 4.20±0.04 s | 3.14±0.01 x | 5.52±0.02 m | *4.29±1.03 Bb* |
| ***Average of tillage methods*** | *Chisel* | *7.78±2.33 Ab*^***^ | *4.60±0.50 Ba* | *3.87±0.87 Cc* | *5.42±2.23 b* |
|  | *Disc Harrow* | *9.22±3.76 Aa* | *4.43±1.17 Cb* | *7.68±1.42 Ba* | *7.11±3.10 a* |
|  | *No Tillage* | *3.48±0.56 Bc* | *2.87±0.63 Cc* | *6.24±0.83 Ab* | *4.19±1.63 c* |
| ***Average of fertilizers*** | *Olive Blackwater* | *6.84±3.04 Bc* | *4.28±1.65 Ca* | *7.16±2.08 Aa* | *6.09±2.60 a* |
|  | *Antep Radish* | *7.04±4.04 Ab* | *3.80±1.12 Cb* | *4.79±1.64 Bd* | *5.21±2.85 c* |
|  | *Broccoli* | *7.24±3.81 Aa* | *4.26±0.77 Ca* | *6.59±1.69 Bb* | *6.03±2.69 b* |
|  | *Control* | *6.19±3.65 Ad* | *3.54±0.69 Cc* | *5.18±1.29 Bc* | *4.97±2.45 d* |
|  |  |  |  |  |  |
|  |  |  |  |  |  |
| **Peonidin-3-*O*-glucoside (%)** | | | | | |
| **Tillage** | **Fertilizer** | **Years** | | | ***Average of years*** |
|  |  | **2020** | **2021** | **2022** |  |
| **Chisel** | Olive Blackwater | 40.07±0.30 j^*^ | 14.40±0.15 p-r | 14.56±0.24 p-r | *23.01±12.80 Cb*^**^ |
|  | Antep Radish | 18.66±0.14 o | 11.81±0.09 st | 10.38±0.07 u | *13.62±3.83 Cd* |
|  | Broccoli | 22.30±0.23 m | 14.11±0.15 qr | 21.02±0.55 n | *19.14±3.83 Cc* |
|  | Control | 63.87±0.67 e | 11.58±0.09 st | 12.08±0.12 st | *29.18±26.03 Ca* |
| **Disc Harrow** | Olive Blackwater | 52.39±0.39 i | 13.83±0.15 r | 85.05±1.13 b | *50.42±30.88 Ab* |
|  | Antep Radish | 62.60±0.66 f | 11.34±0.08 t | 61.94±0.70 f | *45.29±25.47 Ac* |
|  | Broccoli | 62.05±0.46 f | 32.53±0.24 k | 69.93±1.42 d | *54.84±17.09 Aa* |
|  | Control | 15.30±0.16 p | 28.44±0.30 l | 55.69±0.42 h | *33.14±17.84 Ad* |
| **No Tillage** | Olive Blackwater | 12.55±0.10 s | 8.87±0.10 v | 89.50±1.19 a | *36.97±39.43 Bb* |
|  | Antep Radish | 14.99±0.16 pq | 18.97±0.20 o | 64.56±0.72 e | *32.84±23.86 Bc* |
|  | Broccoli | 12.30±0.10 st | 33.13±0.35 k | 74.80±1.52 c | *40.08±27.57 Ba* |
|  | Control | 14.69±0.15 p-r | 14.27±0.08 qr | 60.14±0.45 g | *29.70±22.83 Bd* |
| ***Average of tillage methods*** | *Chisel* | *36.23±18.70 Ab*^***^ | *12.98±1.35 Cc* | *14.51±4.23 Bc* | *21.24±15.23 c* |
|  | *Disc Harrow* | *48.08±20.22 Ba* | *21.53±9.51 Ca* | *68.15±11.50 Ab* | *45.92±23.95 a* |
|  | *No Tillage* | *13.63±1.28 Cc* | *18.81±9.41 Bb* | *72.25±11.83 Aa* | *34.90±28.19 b* |
| ***Average of fertilizers*** | *Olive Blackwater* | *35.00±17.67 Ba* | *12.37±2.64 Cd* | *63.04±36.42 Aa* | *36.80±30.86 b* |
|  | *Antep Radish* | *32.08±22.94 Bb* | *14.04±3.70 Cc* | *45.63±26.46 Ac* | *30.58±23.57 c* |
|  | *Broccoli* | *32.21±22.79 Bb* | *26.59±9.37 Ca* | *55.25±25.78 Ab* | *38.02±23.47 a* |
|  | *Control* | *31.29±24.44 Bc* | *18.09±7.85 Cb* | *42.63±23.00 Ad* | *30.67±21.68 c* |
| ^*^Different lowercase letters indicate differences according to the tillage × fertilizer × year interaction (P<0.05). ^**^Different lowercase letters indicate differences among fertilizer means within the same soil tillage method, whereas different uppercase letters indicate differences of the same fertilizer across different soil tillage methods (P<0.05). ^***^Different lowercase letters indicate differences among means in the same column, whereas different uppercase letters indicate differences among means in the same row (P<0.05).  Data are presented as mean + standard deviation (SD).  **Peonidin-3-*O*-glucoside acetyl (%)** | | | | | |
| **Tillage** | **Fertilizer** | **Years** | | | ***Average of years*** |
|  |  | **2020** | **2021** | **2022** |  |
| **Chisel** | Olive Blackwater | 69.46±0.73 e^*^ | 38.50±0.21 m | 38.81±0.17 lm | *48.92±15.41 Bb*^**^ |
|  | Antep Radish | 36.71±0.38 n | 27.21±0.28 t | 18.58±0.20 y | *27.50±7.86 Cd* |
|  | Broccoli | 57.76±0.32 g | 42.82±0.23 k | 37.19±0.16 n | *45.92±9.21 Bc* |
|  | Control | 113.26±0.62 b | 30.26±0.32 r | 24.52±0.29 u | *56.01±43.01 Aa* |
| **Disc Harrow** | Olive Blackwater | 80.04±0.84 d | 47.61±0.26 h | 80.32±0.34 d | *69.33±16.29 Aa* |
|  | Antep Radish | 125.94±0.69 a | 33.65±0.35 p | 44.60±0.63 j | *68.06±43.67 Ab* |
|  | Broccoli | 104.19±1.09 c | 34.01±0.35 op | 69.45±0.48 e | *69.22±30.40 Aa* |
|  | Control | 28.00±0.16 s | 30.95±0.17 q | 45.29±0.47 i | *34.75±8.02 Bc* |
| **No Tillage** | Olive Blackwater | 19.79±0.21 x | 18.53±0.10 y | 61.19±0.26 f | *33.17±21.02 Cc* |
|  | Antep Radish | 31.14±0.17 q | 23.21±0.13 v | 38.78±0.55 lm | *31.04±6.75 Bd* |
|  | Broccoli | 22.01±0.23 w | 39.24±0.22 lm | 58.13±0.40 g | *39.79±15.65 Ca* |
|  | Control | 34.63±0.19 o | 27.08±0.43 t | 39.34±0.41 l | *33.68±5.37 Cb* |
| ***Average of tillage methods*** | *Chisel* | *69.30±29.21 Ab*^***^ | *34.70±6.53 Bb* | *29.77±8.89 Cc* | *44.59±24.99 b* |
|  | *Disc Harrow* | *84.55±38.09 Aa* | *36.56±6.79 Ca* | *59.92±16.15 Ba* | *60.34±30.78 a* |
|  | *No Tillage* | *26.89±6.44 Bc* | *27.01±8.02 Bc* | *49.36±10.82 Ab* | *34.42±13.60 c* |
| ***Average of fertilizers*** | *Olive Blackwater* | *56.43±27.87 Bd* | *34.88±12.88 Cb* | *60.11±18.00 Aa* | *50.47±22.76 b* |
|  | *Antep Radish* | *64.60±46.08 Aa* | *28.02±4.56 Cd* | *33.99±11.83 Bd* | *42.20±31.13 c* |
|  | *Broccoli* | *61.32±35.69 Ab* | *38.69±3.84 Ca* | *54.92±14.18 Bb* | *51.64±23.51 a* |
|  | *Control* | *58.63±41.07 Ac* | *29.43±1.81 Cc* | *36.39±9.27 Bc* | *41.48±26.60 d* |
| **Malvidin-3-*O*-glucoside (%)** | | | | | |
| **Tillage** | **Fertilizer** | **Years** | | | ***Average of years*** |
|  |  | **2020** | **2021** | **2022** |  |
| **Chisel** | Olive Blackwater | 53.67±0.32 j^*^ | 43.77±0.38 m | 44.26±0.66 m | *47.23±4.85 Bb*^**^ |
|  | Antep Radish | 31.90±0.19 q | 27.34±0.16 t | 18.27±0.11 y | *25.84±6.01 Cc* |
|  | Broccoli | 57.41±0.50 h | 49.20±0.43 l | 41.85±0.41 n | *49.49±6.76 Ba* |
|  | Control | 94.53±0.82 b | 30.73±0.18 r | 24.34±0.06 u | *49.87±33.61 Aa* |
| **Disc Harrow** | Olive Blackwater | 59.04±0.35 g | 55.30±0.48 i | 88.70±1.03 c | *67.68±15.86 Aa* |
|  | Antep Radish | 106.25±0.93 a | 34.54±0.21 p | 49.08±0.09 l | *63.29±32.83 Ab* |
|  | Broccoli | 70.39±0.42 e | 22.74±0.13 v | 83.61±1.55 d | *58.91±27.74 Ac* |
|  | Control | 30.83±0.27 r | 24.02±0.21 u | 50.73±0.31 k | *35.19±12.02 Cd* |
| **No Tillage** | Olive Blackwater | 19.25±0.11 x | 19.19±0.17 x | 70.95±0.82 e | *36.46±25.87 Cb* |
|  | Antep Radish | 34.65±0.30 p | 21.69±0.19 w | 38.89±0.07 o | *31.74±7.76 Bc* |
|  | Broccoli | 21.64±0.13 w | 30.21±0.27 r | 67.99±1.26 f | *39.95±21.36 Ca* |
|  | Control | 38.94±0.34 o | 28.40±0.12 s | 41.65±0.25 n | *36.33±6.06 Bb* |
| ***Average of tillage methods*** | *Chisel* | *59.38±23.52 Ab*^***^ | *37.76±9.42 Ba* | *32.18±11.61 Cc* | *43.11±19.64 b* |
|  | *Disc Harrow* | *66.63±28.24 Ba* | *34.15±13.63 Cb* | *68.03±19.05 Aa* | *56.27±25.98 a* |
|  | *No Tillage* | *28.62±8.73 Bc* | *24.87±4.77 Cc* | *54.87±15.34 Ab* | *36.12±16.98 c* |
| ***Average of fertilizers*** | *Olive Blackwater* | *43.99±18.70 Bd* | *39.42±15.98 Ca* | *67.97±19.39 Aa* | *50.46±21.55 a* |
|  | *Antep Radish* | *57.60±36.51 Aa* | *27.86±5.58 Cc* | *35.42±13.59 Bd* | *40.29±25.34 c* |
|  | *Broccoli* | *49.82±21.87 Bc* | *34.05±11.82 Cb* | *64.48±18.30 Ab* | *49.45±21.30 b* |
|  | *Control* | *54.77±30.03 Ab* | *27.72±2.96 Cc* | *38.91±11.61 Bc* | *40.46±21.20 c* |
| ^*D^ifferent lowercase letters indicate differences according to the tillage × fertilizer × year interaction (P<0.05). ^**^Different lowercase letters indicate differences among fertilizer means within the same soil tillage method, whereas different uppercase letters indicate differences of the same fertilizer across different soil tillage methods (P<0.05). ^***^Different lowercase letters indicate differences among means in the same column, whereas different uppercase letters indicate differences among means in the same row (P<0.05).  Data are presented as mean + standard deviation (SD). | | | | | |
|  |  |  |  |  |  |
| **Malvidin-3-*O*-glucoside acetyl (%)** | | | | | |
| **Tillage** | **Fertilizer** | **Years** | | | ***Average of years*** |
|  |  | **2020** | **2021** | **2022** |  |
| **Chisel** | Olive Blackwater | 54.99±0.90 e^*^ | 23.67±0.25 no | 23.94±0.37 no | *34.20±15.60 Bb*^**^ |
|  | Antep Radish | 32.55±0.53 g | 24.37±0.40 mn | 16.43±0.26 wx | *24.45±6.99 Bd* |
|  | Broccoli | 35.37±0.36 f | 26.49±0.28 l | 22.71±0.21 p | *28.19±5.63 Bc* |
|  | Control | 59.02±0.61 d | 27.27±0.44 k | 21.76±0.28 q | *36.02±17.42 Aa* |
| **Disc Harrow** | Olive Blackwater | 60.77±0.99 c | 29.64±0.31 i | 28.44±0.37 j | *39.62±15.89 Ab* |
|  | Antep Radish | 66.04±0.68 b | 30.52±0.49 h | 22.84±0.24 p | *39.80±19.96 Ab* |
|  | Broccoli | 82.18±1.35 a | 16.43±0.27 wx | 24.83±0.48 m | *41.15±31.00 Aa* |
|  | Control | 16.89±0.17 vw | 11.60±0.27 aa | 23.47±0.38 op | *17.32±5.16 Cc* |
| **No Tillage** | Olive Blackwater | 17.40±0.29 v | 8.90±0.09 cc | 14.37±0.19 z | *13.56±3.74 Cd* |
|  | Antep Radish | 18.90±0.19 u | 9.94±0.10 bb | 15.12±0.29 y | *14.66±3.90 Cc* |
|  | Broccoli | 19.46±0.32 tu | 15.28±0.16 y | 20.53±0.40 rs | *18.42±2.42 Cb* |
|  | Control | 21.15±0.22 qr | 15.71±0.07 xy | 20.13±0.33 st | *19.00±2.51 Ba* |
| ***Average of tillage methods*** | *Chisel* | *45.48±12.18 Ab*^***^ | *25.45±1.57 Ba* | *21.21±3.00 Cb* | *30.71±12.86 b* |
|  | *Disc Harrow* | *56.47±25.26 Aa* | *22.05±8.59 Cb* | *24.90±2.29 Ba* | *34.47±21.81 a* |
|  | *No Tillage* | *19.23±1.42 Ac* | *12.46±3.20 Cc* | *17.54±2.94 Bc* | *16.41±3.89 c* |
| ***Average of fertilizers*** | *Olive Blackwater* | *44.39±20.41 Ab* | *20.73±9.25 Cb* | *22.25±6.23 Bb* | *29.12±16.96 a* |
|  | *Antep Radish* | *39.16±21.01 Ac* | *21.61±9.15 Ba* | *18.13±3.59 Cd* | *26.30±15.92 b* |
|  | *Broccoli* | *45.67±28.24 Aa* | *19.40±5.34 Cc* | *22.69±1.89 Ba* | *29.25±19.93 a* |
|  | *Control* | *32.35±20.08 Ad* | *18.19±7.04 Cd* | *21.79±1.47 Bc* | *24.11±13.33 c* |
|  |  |  |  |  |  |
|  |  |  |  |  |  |
| **Malvidin-3-*O*-(-*p*-coumaryl)-glucoside (%)** | | | | | |
| **Tillage** | **Fertilizer** | **Years** | | | ***Average of years*** |
|  |  | **2020** | **2021** | **2022** |  |
| **Chisel** | Olive Blackwater | 5.56±0.03 g^*^ | 2.33±0.01 m-p | 2.35±0.02 m-o | *3.42±1.61 Cb*^**^ |
|  | Antep Radish | 16.64±0.36 a | 1.89±0.02 st | 1.66±0.02 u | *6.73±7.44 Ba* |
|  | Broccoli | 5.56±0.03 g | 2.28±0.01 n-p | 2.55±0.02 l | *3.47±1.58 Bb* |
|  | Control | 6.09±0.03 e | 1.86±0.03 t | 1.94±0.03 st | *3.29±2.10 Ac* |
| **Disc Harrow** | Olive Blackwater | 9.69±0.17 b | 2.24±0.01 o-q | 3.77±0.03 h | *5.23±3.41 Ab* |
|  | Antep Radish | 16.64±0.36 a | 1.82±0.03 tu | 2.40±0.04 l-o | *6.96±7.27 Aa* |
|  | Broccoli | 5.85±0.03 f | 2.51±0.01 lm | 2.88±0.04 k | *3.75±1.59 Ac* |
|  | Control | 2.47±0.01 l-n | 3.09±0.06 j | 2.15±0.03 p-r | *2.57±0.41 Bd* |
| **No Tillage** | Olive Blackwater | 2.01±0.03 r-t | 9.05±0.20 d | 2.39±0.02 l-o | *4.48±3.43 Bb* |
|  | Antep Radish | 2.42±0.01 l-o | 9.33±0.20 c | 2.06±0.08 q-s | *4.60±3.55 Ca* |
|  | Broccoli | 1.97±0.03 r-t | 3.45±0.02 i | 3.10±0.04 j | *2.84±0.67 Cc* |
|  | Control | 2.38±0.01 l-o | 2.30±0.01 n-p | 2.41±0.03 l-o | *2.36±0.05 Cd* |
| ***Average of tillage methods*** | *Chisel* | *8.47±4.94 Ab*^***^ | *2.09±0.23 Bc* | *2.13±0.36 Bc* | *4.23±4.12 b* |
|  | *Disc Harrow* | *8.67±5.50 Aa* | *2.41±0.48 Cb* | *2.80±0.64 Ba* | *4.63±4.26 a* |
|  | *No Tillage* | *2.20±0.22 Cc* | *6.03±3.33 Aa* | *2.49±0.40 Bb* | *3.57±2.58 c* |
| ***Average of fertilizers*** | *Olive Blackwater* | *5.76±3.33 Ab* | *4.54±3.39 Ba* | *2.84±0.70 Ca* | *4.38±2.93 b* |
|  | *Antep Radish* | *11.90±7.11 Aa* | *4.35±3.74 Bb* | *2.04±0.32 Cc* | *6.10±6.19 a* |
|  | *Broccoli* | *4.46±1.87 Ac* | *2.75±0.54 Cc* | *2.85±0.24 Ba* | *3.35±1.35 c* |
|  | *Control* | *3.65±1.83 Ad* | *2.41±0.54 Bd* | *2.17±0.21 Cb* | *2.74±1.25 d* |

^*^Different lowercase letters indicate differences according to the tillage × fertilizer × year interaction (P<0.05). ^**^Different lowercase letters indicate differences among fertilizer means within the same soil tillage method, whereas different uppercase letters indicate differences of the same fertilizer across different soil tillage methods (P<0.05). ^***^Different lowercase letters indicate differences among means in the same column, whereas different uppercase letters indicate differences among means in the same row (P<0.05).

Data are presented as mean + standard deviation (SD).
